# An Alternative Approach towards C-12 Functionalized Scalaranic Sesterterpenoids Synthesis of 17-Oxo-20-norscalaran-12α,19-*O*-lactone †

**DOI:** 10.3390/md19110636

**Published:** 2021-11-12

**Authors:** Olga Morarescu, Marina Grinco, Veaceslav Kulciţki, Sergiu Shova, Nicon Ungur

**Affiliations:** 1 Laboratory of Chemistry of Natural and Biologically Active Compounds, Institute of Chemistry, 3 Academiei Str., MD 2028 Chişinău, Moldova; olgamorarescu7@gmail.com (O.M.); grinkom@yahoo.com (M.G.); kulcitki@yahoo.com (V.K.); 2 CEEC Institute, Ningbo University of Technology, No. 201, Fenghua Road, Ningbo City 315211, China; shova@icmpp.ro; 3 Department of Inorganic Polymers, “Petru Poni” Institute of Macromolecular Chemistry, 41A Aleea Gr. Ghica Voda, 700487 Iasi, Romania

**Keywords:** scalarane sesterterpenoids, synthesis, natural terpenoids, X-ray analysis

## Abstract

Scalarane sesterterpenoids emerged as interesting bioactive natural products which were isolated extensively from marine sponges and shell-less mollusks. Some representatives were also reported recently from superior plants. Many scalarane sesterterpenoids displayed a wide spectrum of valuable properties, such as antifeedant, antimicrobial, antifungal, antitubercular, antitumor, anti-HIV properties, cytotoxicity and stimulation of nerve growth factor synthesis, as well as anti-inflammatory activity. Due to their important biological properties, many efforts have been undertaken towards the chemical synthesis of natural scalaranes. The main synthetic challenges are connected to their complex polycyclic framework, chiral centers and different functional groups, in particular the oxygenated functional groups at the C-12 position, which are prerequisites of the biological activity of many investigated scalaranes. The current work addresses this problem and the synthesis of 17-oxo-20-norscalaran-12α,19-*O*-lactone is described. It was performed via the 12α-hydroxy-*ent*-isocopal-13(14)-en-15-al obtained from (-)-sclareol as an accessible starting material. The tetracyclic lactone framework was built following an addition strategy, which includes the intramolecular Michael addition of a diterpenic acetoacetic ester and an intramolecular aldol condensation reaction as key synthetic steps. The structure and stereochemistry of the target compound have been proven by X-Ray diffraction method.

## 1. Introduction

**Figure 1:**
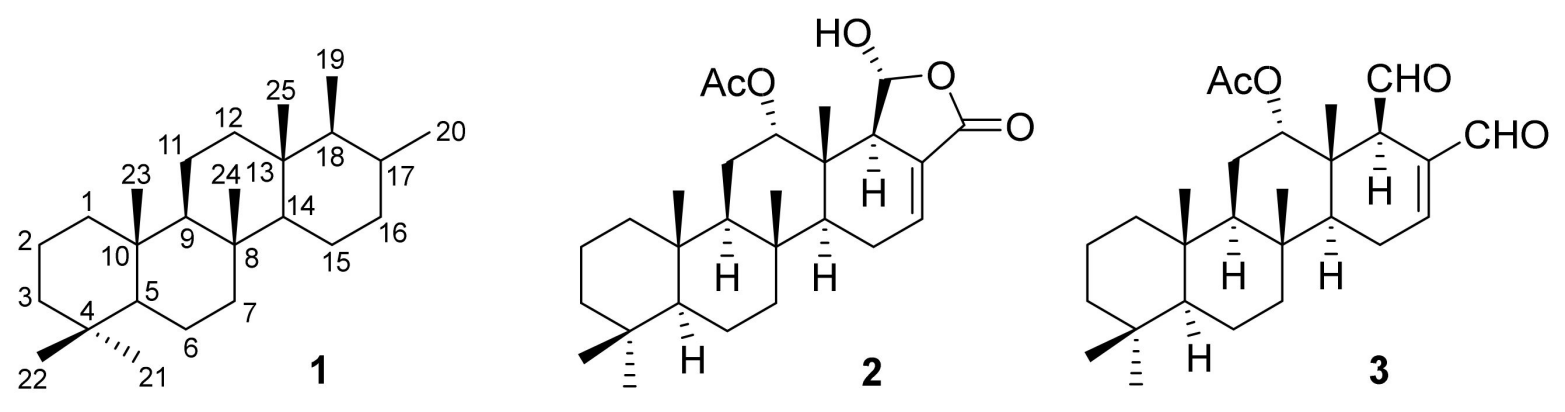
Representatives of the scalaranic sesterterpenoids.

Scalaranic sesterterpenoids are natural products with a tetracyclic carbon skeleton **1** (Figure 1). The first representatives of this terpenoids subclass were isolated in the beginning of the 1970s. In particular, scalarine (**2**) was isolated by Ernesto Fattorusso and collaborators from the see sponges *Cacospongia scalaris* [1], collected in the Mediteranian Sea. Soon after, Guido Cimino and collaborators identified the bioactive sesterterpenoid (-)-scalaradial (**3**) in the extract of another sea sponge, *Cacospongia mollior* [2].

Marine organisms such as sponges or mollusks represent the main source for the scalaranic sesterterpenoids’ isolation [3,4,5,6]. Some recent works report their isolation also from terrestrial plants [7] and fungi [8], thus keeping the focus and the scientific interest towards such compounds. The last 5 years witnessed more than 30 high impact publications connected to scalaranes [9]. This is mainly due to the wide range of their biological activities, including antifeedant, antimicrobial, antifungal, anti-HIV properties, cytotoxicity and anti-inflammatory activity, etc. [3,8]. However, a broader investigation of scalaranes in medicinal chemistry studies is still hampered by their relative scarcity in natural sources and a lot of efforts have been put on the elaboration of pathways for their target synthesis [10]. The structural complexity of the scalarane architecture is connected to their polycyclic backbone, stereochemical issues and specific oxygenations. While the first two challenges have been addressed successfully in several synthetic strategies, the introduction of oxygenated functional groups, especially in the C-12 position of the tetracyclic system still represents a relevant synthetic hurdle. Only few works on the synthesis of cycle B- [11] and C-functionalized [12,13,14,15,16] scalaranes have been reported since 2004. In particular, previous successful reports on the synthesis of C-12—functionalized scalaranes make use of *ent*-isocopalic compounds and assemble the D-cycle via a Diels-Alder cycloaddition approach [12,13,14,15] or employing an intramolecular Heck reaction of tricyclic cheilanthanes [16]. The most successful example [15] demonstrates the synthesis of the C-12 functionalized scalaranic framework over 18 synthetic steps with an 4.5% overall yield. We present in the current paper an alternative synthetic rout towards the scalaranes functionalized at the C-12 position.

## 2. Results and Discussion

**Scheme 1:**
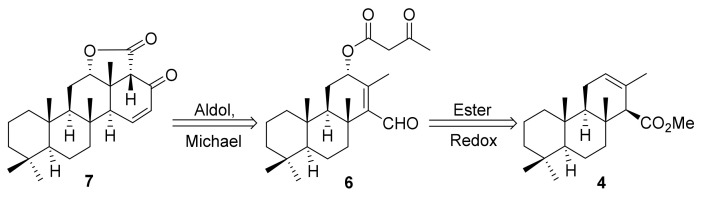
The retrosynthetic scheme towards C-12–functionalized scalaranic framework.

In order to elaborate an alternative strategic approach for the synthesis of a C-12-functionalized tetracyclic framework, we addressed a synthetic pathway basing on the readily available methyl *ent*-isocopalate (**4**) as a convenient chiral building block which can be prepared easily from the commercial (-)-sclareol (**5**). It can be further oxygenated at the C-12 position and homologated with a C-4 fragment in the form of the acetoacetate ester **6** as a pre-requisite of an intramolecular sequence of a Michael–aldol reactions, leading to the closure of the D-cycle in lactone **7** with the required *trans*-stereochemistry (Scheme 1).

The lactone **7** is a valuable intermediate to access highly functionalized scalaranes on flexible manipulation of its functional groups. In our hands, the hydrogenation of the double bond delivered the 17-oxo-20-norscalaran-12α,19-O-lactone (**8**).

**Scheme 2:**
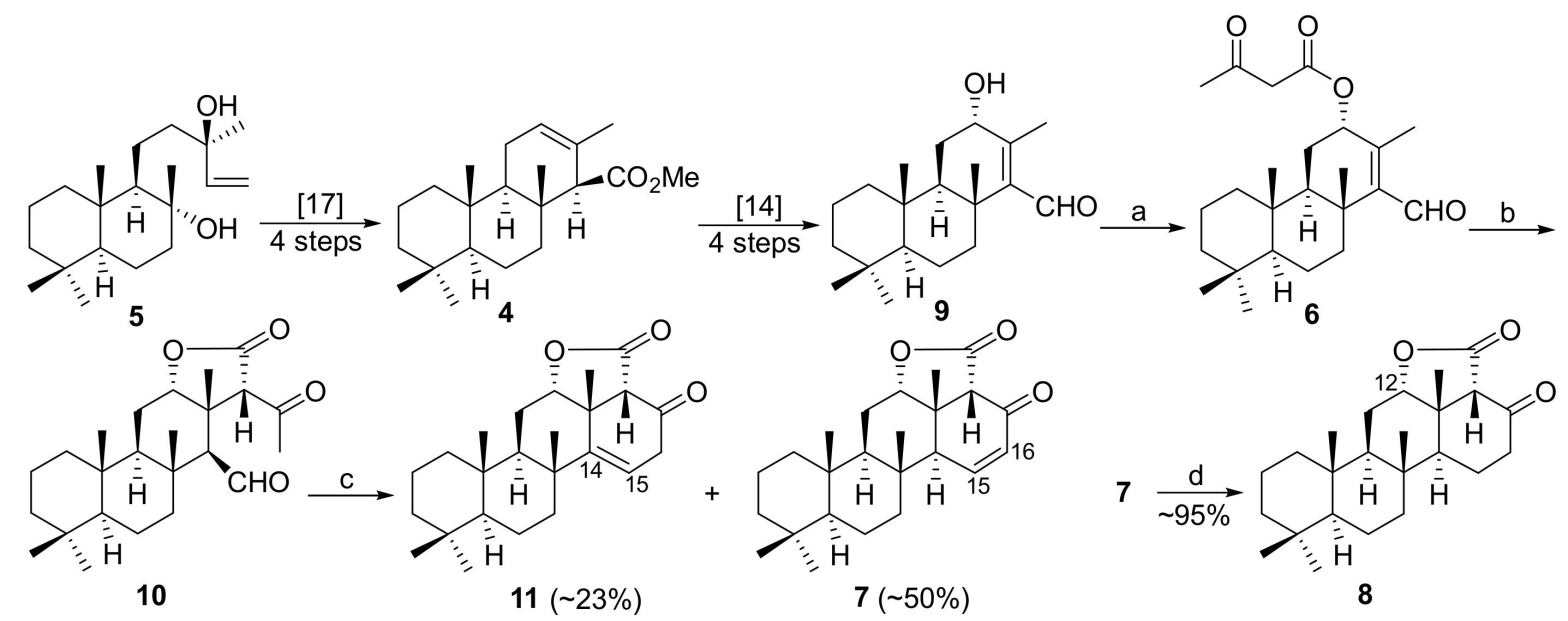
Reagents and conditions: (**a**) Diketene, CH_2_Cl_2_, Et_3_N, 0 °C, 2 h; (**b**) Cs_2_CO_3_, MeCN, reflux, 2 h, ~61% over 2 steps; (**c**) *p*-TsOH, PhH, reflux, 3 h; (**d**) H_2_, 10% Pd/C, EtOAc, 4 h.

Implementation of the planned synthetic strategy was straightforward (Scheme 2). The isocopalic hydroxyaldehyde (**9**) obtained by a known sequence of transformations from **5** via **4** [14,17] was esterified with diketene under mild conditions in dichloromethane, according to the method [18].

The ester **6** resulted in a good yield, and due to its instability was submitted to the next step without purification. The Michael reaction was initiated on immediate treatment of crude ester **6** with caesium carbonate in acetonitrile [19]. The desired lactone **10** was obtained with a good yield (~61% over two steps) and its structure was demonstrated basing on spectral data.

**Figure 2:**
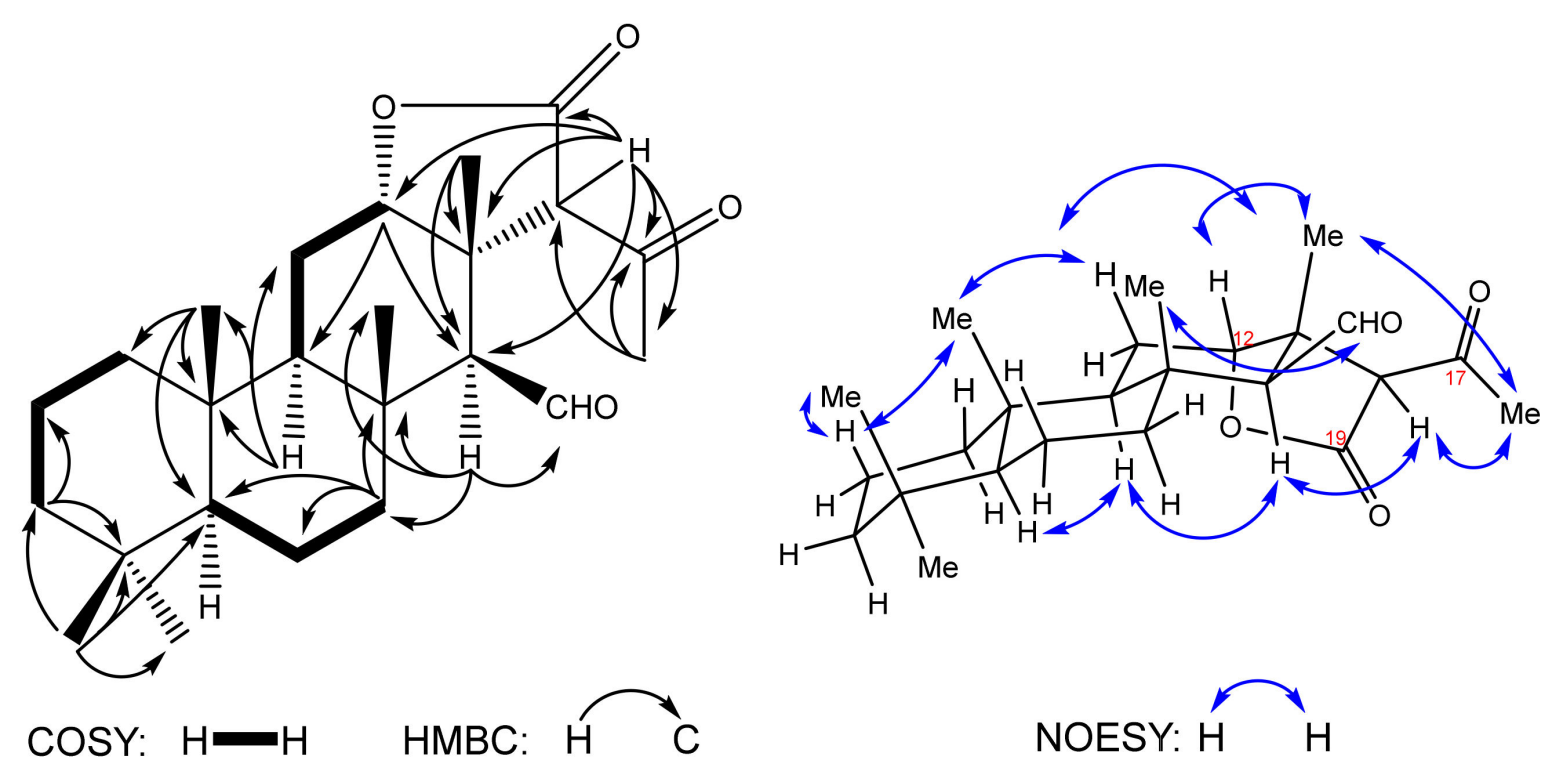
Selected ^1^H-^13^C HMBC, ^1^H-^1^H COSY and NOESY correlations for compound (**10**).

The IR spectrum of compound **10** shows the presence of the aliphatic C-H bonds (2922 cm^−1^) and carbonyl groups (1774, 1711 cm^−1^). The ^13^C spectrum shows peaks of 24 carbons: 6 methyl and 6 methine carbons, 6 methylenic carbons, an oxymethine (δ_C_ 84.4), aldehyde (δ_C_ 204.6) and 6 quaternary carbons, including two carbonyls (δ_C_ 172.8, 203.2). Attribution of ^13^C peaks and assignment of all protons chemical shifts was performed on the basis of 2D HSQC, HMBC and ^1^H-^1^H COSY correlations. In particular, ^1^H and ^13^C NMR signals of six methyl groups at δ_H_ 0.86 (3H-21)/δ_C_ 33.2 (C-21), 0.82 (3H-22)/21.3 (C-22), 0.86 (3H-23)/15.9 (C-23), 1.21 (3H-24)/19.0 (C-24), 1.22 (3H-25)/15.5 (C-25) have been attributed basing on HMBC correlations, along with the methyl adjacent to the keto group found at δ_H_ 2.34 (H-16)/δ_C_ 33.3 (C-16) (Figure 2). The triplet of the oxymethine proton is detected at δ_H_ 4.63 (t, 2.9, H-12)/δ_C_ 84.4 (C-12) and the doublet of the aldehyde proton at δ_H_ 10.02 (d, 1.4, C-15)/δ_C_ 204.6 [C-15(CHO)]. The methine protons are confirmed at δ_H_ 0.93 (m, H-5), 1.28 (m, H-9), 1.86 (bs, H-14) and 3.93 (s, H-18) by HSQC cross peaks with carbons at δ_C_ 56.2 (C-5), 49.7 (C-9), 65.2 (C-14) and 66.5 (C-18), respectively. HMBC correlations H-18→C-12, C-13, C-14 (Figure 2) confirm the formation of the new bond after the Michael reaction leading to the α-lactone cycle and the pendant methyl ketone.

The relative stereochemistry was established on the basis of the NOESY spectrum (Figure 2). The configuration of the 12β-H proton which corresponds to the starting substrate **6** was confirmed by H-12↔H3-25 correlations. The β-orientation of the aldehyde group is proven by correlations H-14↔H-9 and H-15(CHO)↔H3-24.

The intramolecular aldol reaction of ketoaldehyde **10** was triggered upon treatment with PTSA. The cyclization occurred with a good yield and selectivity; the desired unsaturated ketolactone **7** predominated over its isomer **11**, which was formed as a result of double bond migration under acidic reaction conditions. Such isomerizations are known in aldol-related cyclizations; we did not make any attempts to optimize this transformation.

**Figure 3:**
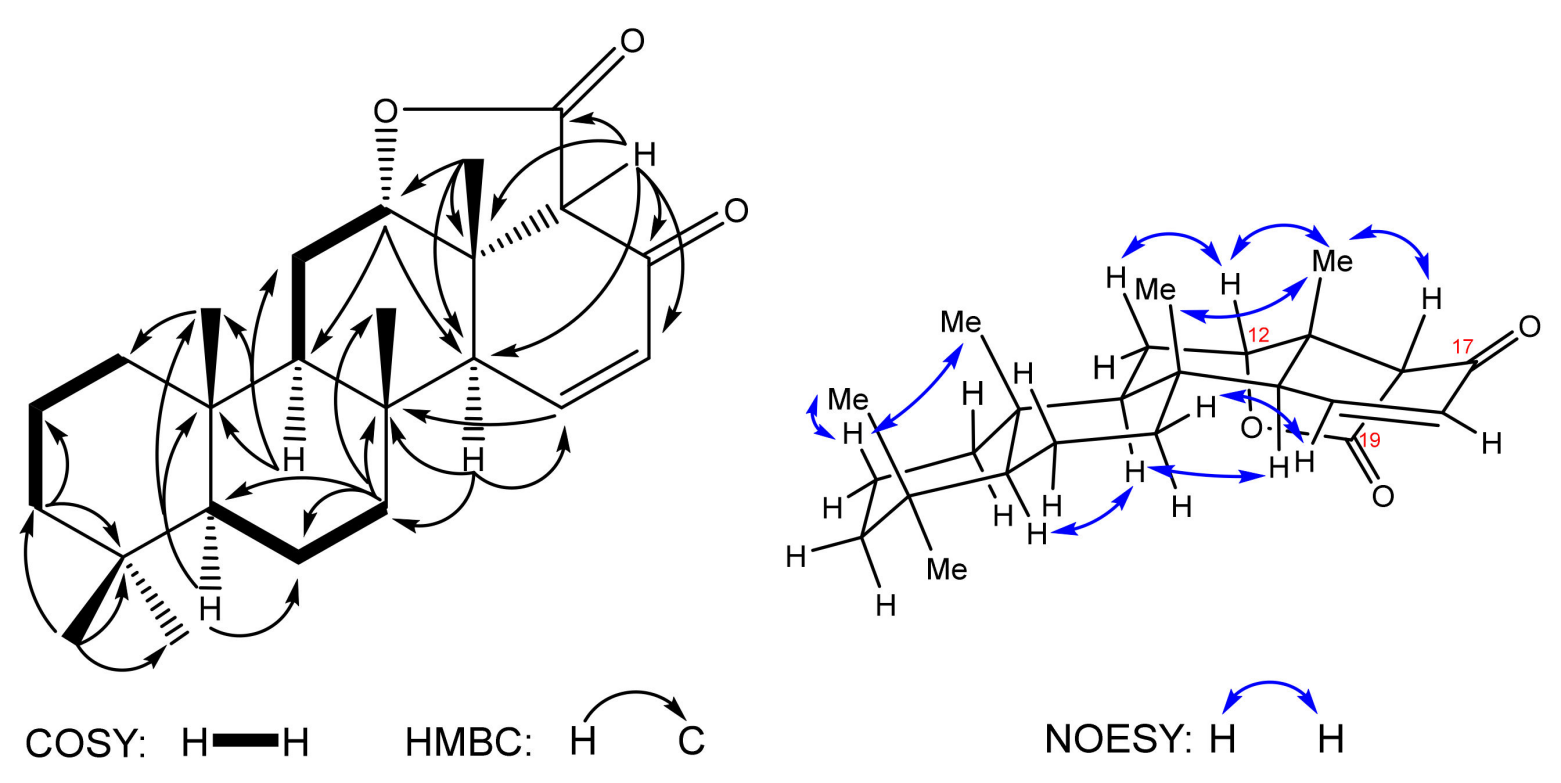
Selected ^1^H-^13^C HMBC, ^1^H-^1^H COSY and NOESY correlations for compound **7**.

The IR spectrum of compound **7** shows the presence of the aliphatic C-H bonds (2920, 2865 cm^−1^) and carbonyl group (1760 cm^−1^). The structure of compound **7** was elucidated on the basis of NMR spectral data, in particular of 2D HSQC, HMBC and ^1^H-^1^H COSY correlations (Figure 3).

The ^1^H and ^13^C NMR show neither aldehyde group nor methyl ketone specific signals, whereas a double bond is clearly detected (δ_C_ 129.9, 149.5). In this line, the ^1^H-^1^H COSY cross peaks corresponding to H-15↔H-16↔H-17 correlations show convincingly the D-ring closure as a result of the intramolecular aldol reaction in the substrate **8**. On the basis of ^13^C and HSQC spectra, the carbon backbone of compound **7** is revealed to include 24 carbon atoms: 5 methyl, 6 methylene groups and 7 methine groups, 6 quaternary carbons, including two carbonyls (δ_C_ 169.7, 188.6). Attribution of ^13^C peaks and assignment of all protons chemical shifts resulted in five methyls at δ_H_ 0.85 (3H-21)/δ_C_ 33.2 (C-21), 0.82 (3H-22)/21.3 (C-22), 0.86 (3H-23)/15.6 (C-23), 0.99 (3H-24)/18.0 (C-24) and 1.27 (3H-25)/18.4 (C-25). The methine protons are confirmed at δ_H_ 0.89 (m, H-5), 1.32 (m, H-9), 4.38 (t, 2.8, H-12), 2.39 (t, 3, H-14), 3.07 (s, H-18) by HSQC cross peaks with carbons at δ_C_ 56.6 (C-5), 52.2 (C-9), 82.8 (C-12), 50.4 (C-14) and 64.9 (C-18), respectively. The protons attached to sp^2^ carbons are detected at δ_H_ 7.09 (dd, 10, 3, H-15)/δ_C_ 149.5 (C-15) and 6.09 (dd, 10, 3, H-16)/129.9 (C-16).

The careful examination of 2D NMR confirmed assembling of the pentacyclic system including tetracyclic nor-scalaranic framework condensed with the C-12–C-18 lactone ring and oxygenated at C-17 with the keto group. The relative stereochemistry of lactone **7** was established on the basis of NOESY spectrum (Figure 3). Correlation H-12↔H3-25↔H-18 clearly shows the α-orientation of the lactone ring, and H-14 α-orientation is proven by H-14↔H-9 correlation.

The spectral data of minor lactone **11** are very much similar to those of major compound **7**. The only major difference represents the double bond position in cycle D, which is trisubstituted and placed at C-14–C-15 carbon atoms.

**Figure 4:**
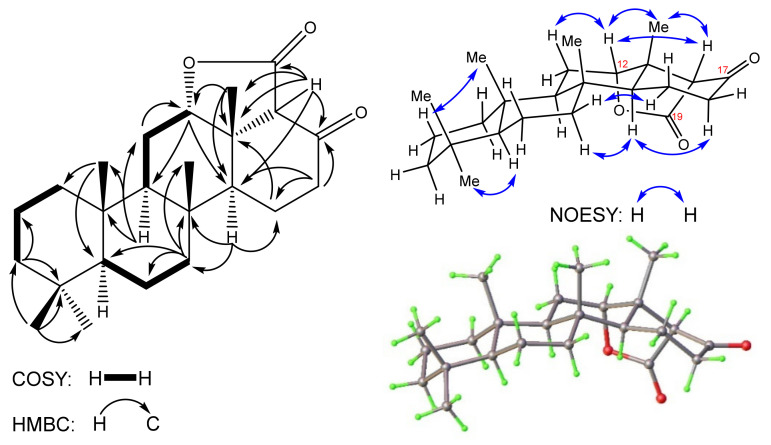
Selected ^1^H-^13^C HMBC, ^1^H-^1^H COSY and NOESY correlations and X-ray molecular structure of compound **8**.

The major pentacyclic ketolactone **7** represents a very useful compound for a flexible generation of a whole array of molecular diversity. Direct short range functionalizations are feasible in cycles C and D, and, evidently, olefination of the C-17 keto group can provide the C-25—scalaranic backbone. In order to finally prove the relative stereochemistry of lactone **7**, we performed X-ray analysis of its hydrogenation product **8**, which turned out to provide suitable crystals for this investigation. The hydrogenation of **7** went smoothly (95%) after treatment with palladium under hydrogen gas atmosphere. The spectral data of saturated ketolactone **8** have shown a perfect match to its suggested stereochemistry. In particular, 2D NMR experiments confirmed the structural changes of the substrate **7**, consisting of the modified chemical shift values for C-15 and C-16 positions to δ_H_ 1.96–1.63 (m, 2H-15)/δ_C_ 18.5 (C-15) and 2.58–2.36 (m, 2H-16)/48.8 (C-16). Compound **8** shows a total of 24 carbon atoms, including 6 methyl, 8 methylene and 5 methine groups, and 6 quaternary carbons. Attribution of ^13^C peaks and assignment of all protons chemical shifts show methyl groups at δ_H_ 0.85 (3H-21)/δ_C_ 33.2 (C-21), 0.82 (3H-22)/21.3 (C-22), 0.85 (3H-23)/15.8 (C-23), 0.90 (3H-24)/17.0 (C-24) and 1.25 (3H-25)/18.4 (C-25). The attribution of C-H groups included signals at δ_H_ 0.89 (m, H-5), 1.34 (m, H-9), 4.29 (t, 2.8, H-12), 1.54 (m, H-14), 3.05 (s, H-18), which correspond to carbon atoms at δ_C_ 56.4 (C-5), 52.4 (C-9), 84.2 (C-12), 50.4 (C-14) and 67.5 (C-18). NOESY correlations for compound **8** confirm the desired *trans*-stereochemistry between newly built cycles of the tetracyclic scalaranic framework (Figure 4).

The chemical composition and crystal structure of compound **8** were confirmed by single crystal X-ray diffraction. A single crystal of ketolactone **8** was obtained on its crystallization from ethyl acetate-diethyl ether solvent mixture (1:1). According to X-ray crystallography, compound **8** exhibits a molecular crystal structure crystallizing the P212121 Shohnke space group of the orthorhombic system with one neutral entity in the asymmetric part, as shown in Figure 4. In the crystal, the neutral molecules are interacting through C-H**···**O hydrogen bonding to form infinite supramolecular ribbons running along an axis. A detailed report on the X-ray experiment, including one-dimensional architecture and crystal packing, is available as Supplementary Materials.

## 3. Materials and Methods

### 3.1. General Experimental Procedures

Melting points were measured with a Boethius heating stage. Optical rotations: Jasco-DIP-370 polarimeter; 5 cm cell; in CHCl_3_. IR Spectra: Spectrum-100 FT-IR spectrophotometer (PerkinElmer), with the universal ATR sampling accessory; ν in cm^−1^. ^1^H- and ^13^C-NMR Spectra: Bruker-Avance-III spectrometer (400.13 and 100.61 MHz); in CDCl_3_; δ in ppm rel. to CHCl_3_ as internal standard (δ_H_ 7.26 and δ_C_ 77.0), *J* in Hz. The carbon and hydrogen content of compounds were determined by standard microanalysis on Vario-EL-III-CHNOS Elemental Analyzer. Commercial Merck silica gel 60 (70–230 mesh ASTM) was used for flash chromatography and Merck pre-coated silica gel plates were used for TLC. The chromatograms were sprayed with 0.1% solution of cerium (IV) sulfate in 2N sulfuric acid, and heated at 80 °C for 5 min to detect the spots. Treatment of reaction mixtures in organic solvents included the extraction by diethyl ether, washing of the extract with water up to neutral reaction, drying over anhydrous Na_2_SO_4_, filtering and solvent removal in vacuum.

### 3.2. Single Crystal X-Ray Diffraction

X-ray diffraction measurements were carried out with a Rigaku Oxford-Diffraction XCALIBUR E CCD diffractometer equipped with graphite-monochromated MoKα radiation. A single crystal was positioned at 40 mm from the detector and 201 frames were measured each for 125 s over 1° scan width. The unit cell determination and data integration were carried out using the CrysAlis package of Oxford Diffraction [20]. The structures were solved by Intrinsic Phasing using Olex2 [21] software with the SHELXT [22] structure solution program, and refined by full-matrix least-squares on *F^2^* with SHELXL-2015 [23] using an anisotropic model for non-hydrogen atoms. In the absence of significant anomalous scattering, the absolute configuration of the structures could not be reliably determined. Friedel pairs were merged and any references to the Flack parameter were removed. The H atoms were placed geometrically and constrained to ride on their parent atoms with d_CH_ = 0.96 Å and Uiso values of 1.2 Ueq of the parent atoms. The crystallographic data and refinement details are quoted in Table S1, whereas bond lengths and angles are given in Table S2 (Supplementary Materials available).

### 3.3. 12α-Hydroxy-ent-isocopal-13,14-en-15-al (***9***)

Compound **9** was obtained according to the described method [14]. 12α-Hydroxy-ent-isocopal-13,14-en-15-al (**9**) was obtained as a white crystalline solid. Mp: 123–125 °C; (Lit. [13] Mp: 134–135 °C); $\alpha_{D}^{20}\;$–69.8 (c 0.31, CHCl_3_). IR (υ, cm^−1^): 3388, 2869, 1678, 1456, 1379, 1042, 733. ^1^H (400.13 MHz, CDCl_3_) δ_H_: 0.81 (3H, s, H-19), 0.83 (3H, s, H-18), 0.87 (3H, s, H-20), 1.16 (3H, s, H-17), 2.12 (3H, s, H-16), 4.04 (1H, dd, *J* = 4.5, 1.2 Hz, H-12), 10.08 (1H, s, CHO). ^13^C (100.61 MHz, CDCl_3_) δ_C_: 16.5 (q, C-20), 16.8 (q, C-16), 18.4 (t, C-2), 18.5 (t, C-6), 19.8 (q, C-17), 21.2 (q, C-19), 27.1 (t, C-11), 33.2 (q, C-18), 33.2 (s, C-4), 37.0 (s, C-10), 37.6 (t, C-7), 38.9 (s, C-8), 39.6 (t, C-1), 42.0 (t, C-3), 50.3 (d, C-9), 56.5 (d, C-5), 70.8 (d, C-12), 145.1 (d, C-14), 148.1 (s, C-13), 194.3 (s, C-15).

### 3.4. Synthesis of Compound ***6***

Et_3_N (80 μL, 0.57 mmol) and diketene (45 μL, 0.57 mmol) were added to a solution of hydroxyaldehyde **9** (117 mg, 0.38 mmol) and benzene (2 mL) in the inert atmosphere. The reaction mixture was stirred for 30 min at 0 °C and 2 h at room temperature. After the usual work-up, the extract was dried and filtered. The solvent was removed under reduced pressure and the residue (~156 mg) of compound **6** was obtained, pale yellow viscous oil. Because the substance **6** is unstable, it was used in the next step without any purification. ^1^H (400.13 MHz, CDCl_3_) δ_H_: 1.17 (3H, s, Me-24), 1.97 (3H, s, Me-25), 2.27 (3H, s, Me-16), 3.51 (2H, s, H-18), 5.31 (1H, bd, *J* = 3.9 Hz, H-12), 10.08 [1H, s, C-15(CHO)]; ^13^C (100.61 MHz, CDCl_3_) δ_C_: 16.2 (q, C-23), 16.2 (q, C-25), 18.4 (t, C-2), 18.4 (t, C-6), 19.9 (q, C-24), 21.2 (q, C-22), 33.2 (s, C-4), 36.9 (s, C-10), 37.5 (t, C-7), 38.5 (s, C-8), 39.4 (t, C-1), 41.9 (t, C-3), 50.3 (t, C-18), 50.5 (d, C-9), 56.2 (d, C-5), 73.8 (d, C-12), 143.0 (s, C-14), 147.39 (s, C-13), 166.7 (s, C-19), 193.9 (s, C-15), 200.0 (s, C-17).

### 3.5. Synthesis of Compound ***10***

To a solution of compound **6** (140 mg, 0.36 mmol) and anhydrous MeCN, and inert atmosphere, was added anhydrous Cs_2_CO_3_ (122 mg, 0.37 mmol). The reaction mixture was stirred for 15 min at room temperature and 2 h at reflux. After the usual work-up, the extract was dried and filtered. The solvent was removed, and the residue (~146 mg) was purified on a silica gel (5 g) column (petroleum ether–ethyl acetate, gradient elution), resulting in compound **10** (85 mg, ~61% over two steps from **9**), pale yellow viscous oil. $\alpha_{D}^{20\;}$–19.7 (c 0.21, CHCl_3_). IR (ν, cm^−1^): 2922, 2870, 1774, 1711, 1390, 1207. ^1^H (400.13 MHz, CDCl_3_) δ_H_: 0.82 (3H, s, Me-22), 0.86 (6H, s, Me-21, 23), 1.21 (3H, s, Me-24), 1.24 (3H, s, Me-25), 1.86 (1H, bs, H-14) 2.34 (3H, s, Me-16), 3.93 (1H, s, H-18), 4.63 (1H, t, *J* = 2.9 Hz, H-12), 10.02 (1H, d, *J* = 1.4 Hz, CHO). ^13^C (100.61 MHz, CDCl_3_) δ_C_: 15.5 (q, C-25), 15.9 (q, C-23), 17.8 (t, C-6), 18.3 (t, C-2), 19.0 (q, C-24), 20.3 (t, C-11), 21.3 (q, C-22), 33.2 (q, C-21), 33.3 (s, C-4), 33.3 (q, C-16), 37.1 (s, C-10), 37.5 (s, C-8), 39.1 (t, C-1), 41.5 (t, C-7), 41.8 (t, C-3), 44.9 (s, C-13), 49.7 (d, C-9), 56.2 (d, C-5), 65.2 (d, C-14), 66.5 (d, C-18), 84.4 (d, C-12), 172.8 (s, C-19), 203.2 (s, C-17), 204.6 (s, C-15). Anal. Calc. for C_2_4H_36_O_4_: C 74.19, H 9.49; found: C 74.24, H 9.41.

### 3.6. Intramolecular Aldol Condensation of Compound ***10***

To a solution of compound **10** (78 mg, 0.20 mmol) and anhydrous benzene (3 mL), *p*-TsOH (11 mg, 0.06 mmol) was added, and the reaction mixture was refluxed for 4 h. After the usual work-up, the extract was dried and filtered. The solvent was removed, and the residue (~76 mg) was purified on a silica gel (3.5 g) column (petroleum ether—ethyl acetate, gradient elution), resulting in compound **11** (18 mg, 23%) and compound (**7**) (39.1 mg, ~50%).

#### 3.6.1. Compound **11**

Pale yellow amorphous gum. IR (ν, cm^−1^): 2922, 2870, 1774, 1711, 1390, 1207. IR (ν, cm^−1^): 2916, 2872, 1756, 1677, 1142, 1070. ^1^H (400.13 MHz, CDCl_3_) δ_H_: 0.83 (3H, s, Me-22), 0.87 (3H, s, Me-21), 0.90 (3H, s, Me-23), 1.14 (3H, s, Me-24), 1.40 (3H, s, Me-25), 3.19 (1H, s, H-18), 4.42 (1H, t, *J* = 2.8 Hz, H-12), 2.88–2.93 (1H, dd, *J* = 22.7, 2.7 Hz, H-16), 3.10–3.16 (1H, dd, *J* = 22.7, 5.2 Hz, H-16), 5.66 (1H, dd, *J* = 5.1, 2.7 Hz, H-15). ^13^C (100.61 MHz, CDCl_3_) δ_C_: 16.1 (q, C-23), 18.5 (t, C-2), 18.5 (t, C-6), 20.7 (t, C-11), 21.5 (q, C-22), 23.2 (q, C-24), 23.6 (q, C-25), 33.3 (s, C-4), 33.3 (q, C-21), 37.5 (s, C-10), 38.2 (t, C-16), 39.4 (t, C-1), 39.6 (s, C-8), 39.9 (t, C-7), 41.8 (t, C-3), 47.6 (s, C-13), 48.4 (d, C-9), 56.2 (d, C-5), 65.1 (d, C-18), 85.1 (d, C-12), 117.6 (d, C-15), 148.2 (s, C-14), 170.3 (s, C-19), 201.7 (s, C-17). Anal. Calc. for C_24_H_34_O_3_: C 77.80, H 9.25; found: C 77.49, H 9.31.

#### 3.6.2. Compound **7**

White crystalline solid. Mp: 260–262 °C; $\alpha_{D}^{20}$ –14.6 (c 0.19, CHCl_3_). IR (ν, cm^−1^): 2920, 2865, 1760, 1681, 1149, 1077. ^1^H (400.13 MHz, CDCl_3_) δ_H_: 0.82 (3H, s, Me-22), 0.85 (3H, s, Me-21), 0.86 (3H, s, Me-23), 0.99 (3H, s, Me-24), 1.27 (3H, s, Me-25), 2.39 (1H, t, *J* = 3 Hz, H-15), 3.07 (1H, s, H-18), 4.38 (1H, t, *J* = 2.8 Hz, H-12), 6.09 (1H, dd, *J* = 10, 3 Hz, H-16), 7.09 (1H, dd, *J* = 10, 3 Hz, H-15). ^13^C (100.61 MHz, CDCl_3_) δ_C_: 15.6 (q, C-23), 17.7 (t, C-6), 18.0 (q, C-24), 18.4 (t, C-2), 18.4 (q, C-25), 20.1 (t, C-11), 21.3 (q, C-22), 33.2 (q, C-21), 33.3 (s, C-4), 35.6 (s, C-8), 37.0 (s, C-10), 39.1 (t, C-1), 40.1 (t, C-7), 41.8 (t, C-3), 48.0 (s, C-13), 50.4 (d, C-14), 52.2 (d, C-9), 56.6 (d, C-5), 64.9 (d, C-18), 82.8 (d, C-12), 129.9 (d, C-16), 149.5 (d, C-15), 169.7 (s, C-19), 188.6 (s, C-17). Anal. Calc. for C_24_H_34_O_3_: C 77.80, H 9.25; found: C 77.58, H 9.38.

### 3.7. Hydrogenation of Unsaturated Ketone ***7***

To a solution of compound **7** (30 mg, 0.08 mmol) and EtOAc (4 mL), 10% Pd/C (9.4 mg, 0.009 mmol) was added and was stirred for 15 min. Afterwards, a stream of hydrogen was added to the reaction mixture and was stirred for 24 h at room temperature. The mixture was filtered, and the solvent was removed in vacuum. The residue (29 mg) was purified on a silica gel (1.0 g) column (petroleum ether—ethyl acetate, gradient elution), resulting in compound **8** (28,6 mg, ~95%) as a white crystalline solid. Mp: 279–281 °C; $\alpha_{D}^{20}$ 32.2 (c 0.9, CHCl_3_). IR (ν, cm^−1^): 2957, 2923, 2845, 1776, 1712, 1190, 1037. ^1^H (400.13 MHz, CDCl_3_) δ_H_: 0.82 (3H, s, Me-22), 0.85 (3H, s, Me-23), 0.86 (3H, s, Me-22), 0.90 (3H, s, Me-24), 1.25 (3H, s, Me-25), 3.05 (1H, s, H-18), 4.29 (1H, t, *J* = 2.8 Hz, H-12). ^13^C (100.61 MHz, CDCl_3_) δ_C_: 15.8 (q, C-23), 17.0 (q, C-24), 18.1 (t, C-6), 18.4 (t, C-2), 18.4 (q, C-25), 18.5 (t, C-15), 20.5 (t, C-11), 21.3 (q, C-22), 33.2 (q, C-21), 33.3 (s, C-4), 36.9 (s, C-8), 37.1 (s, C-10), 39.3 (t, C-1), 40.8 (t, C-7), 40.8 (t, C-16), 41.9 (t, C-3), 44.9 (s, C-13), 50.4 (d, C-14), 52.5 (d, C-9), 56.4 (d, C-5), 67.5 (d, C-18), 84.2 (d, C-12), 171.0 (s, C-19), 202.4 (s, C-17). Anal. Calc. for C_24_H_36_O_3_: C 77.38, H 9.74; found: C 77.46, H 9.68.

## 4. Conclusions

The target-oriented synthesis of 17-oxo-20-norscalaran-12α,19-O-lactone has been realized, starting from the commercially available (-)-sclareol. The proposed synthetic approach includes 11 steps providing the C-12 functionalized scalaranic framework with the correct *trans*-stereochemistry between C and D cycles with an 11.3% overall yield. The key step constitutes a Michael reaction of a 12α-hydroxy-*ent*-isocopal-13,14-en-15-al acetoacetic ester and its following intramolecular aldol reaction that put in place the tetracyclic scalaranic framework. This simple and efficient strategic pathway represents a new approach towards natural scalaranes with advanced functionalization in both C and D cycles of the tetracyclic skeleton. It opens a broad perspective for structural diversity building in this important natural product family of bioactive compounds.

The structure and stereochemistry of all synthetic intermediates was elucidated on the basis of extensive spectral investigations, including 2D NMR spectroscopy. The stereochemistry of the assembled scalaranic framework was convincingly proven by X-ray monocrystal diffraction studies of the synthesized 17-oxo-20-norscalaran-12α,19-O-lacton.

## Data Availability

Data is contained within the article or Supplementary Materials.

## Supplementary Materials

The following are available online at www.mdpi.com/article/10.3390/md19110636/s1. Figures S1 and S2: X-ray crystal structure report for compound (**8**). Table S1: The crystallographic data and refinement details. Table S2: (a) Bond distances (Å) and (b) angles (°) for compound **8** (CCDC 2116545). ^1^H, ^13^C and 2D NMR spectra of compounds (**6**–**11**).
