# Supplementary material for: An Alternative Approach towards C-12 Functionalized Scalaranic Sesterterpenoids Synthesis of 17-Oxo-20-norscalaran-12α,19-O-lactone
Source: Mar Drugs. 2021 Nov 12;19(11):636. doi: 10.3390/md19110636 (PMC8625711; doi:10.3390/md19110636)
Supplement: Supplementary file 1 [file marinedrugs-19-00636-s001.zip › marinedrugs-1459307-supplementary.pdf]

SUPPLEMENTARY MATERIAL

An alternative approach towards C-12 functionalized scalaranic sesterterpenoids. Synthesis of 17-oxo-20-norscalaran-12α,19-O-lactone

Olga Moraresc u [1], Marina Grinco [1], Veaceslav Kulciţki [1], Sergiu Shova [2,3], Nicon Ungur [1,*]

[1] Institute of Chemistry, 3 Academiei Str., MD 2028 Chişinău, Moldova; olgamoraresc u7@gmail.com> (O.M.); grinkom@yahoo.com, (M.G.); kulcitki@yahoo.com, (V.K.)

[2] Ningbo University of Technology, No. 201, Fenghua Road, Ningbo 315211, China;

[3] "Petru Poni" Institute of Macromolecular Chemistry, 41A Aleea Gr. Ghica Voda, 700487 Iasi, Romania; shova@icmpp.ro (S.S.)

Supplementary data includes:

X-Ray crystal structure report for lactone **8** SM 2

NMR SPECTRA of compounds **6-11** SM8

Reference SM26

Corresponding author:

Prof. Ungur Nicon
Institute of Chemistry
3, Academiei str., Chişinău, MD-2028,
Republic of Moldova
nicon.ungur@gmail.com

X-RAY CRYSTAL STRUCTURE REPORT
Lactone 8 (5187)

EXPERIMENTAL

X-ray diffraction measurements were carried out with a Rigaku Oxford-Diffraction XCALIBUR E CCD diffractometer equipped with graphite-monochromated MoKα radiation. Single crystal was positioned at 40 mm from the detector and 201 frames were measured each for 125 s over 1° scan width. The unit cell determination and data integration were carried out using the CrysAlis package of Oxford Diffraction [1]. The structures were solved by Intrinsic Phasing using Olex2 [2] software with the SHELXT [3] structure solution program and refined by full-matrix least-squares on F^2 with SHELXL-2015 [4] using an anisotropic model for non-hydrogen atoms. In the absence of significant anomalous scattering, the absolute configuration of the structures could not be reliably determined. Friedel pairs were merged and any references to the Flack parameter were removed. The H atoms were placed geometrically and constrained to ride on their parent atoms with d_{CH} = 0.96 Å and Uiso values of 1.2Ueq of the parent atoms. The crystallographic data and refinement details are quoted in Table S1, while bond lengths and angles are given in Table S2.

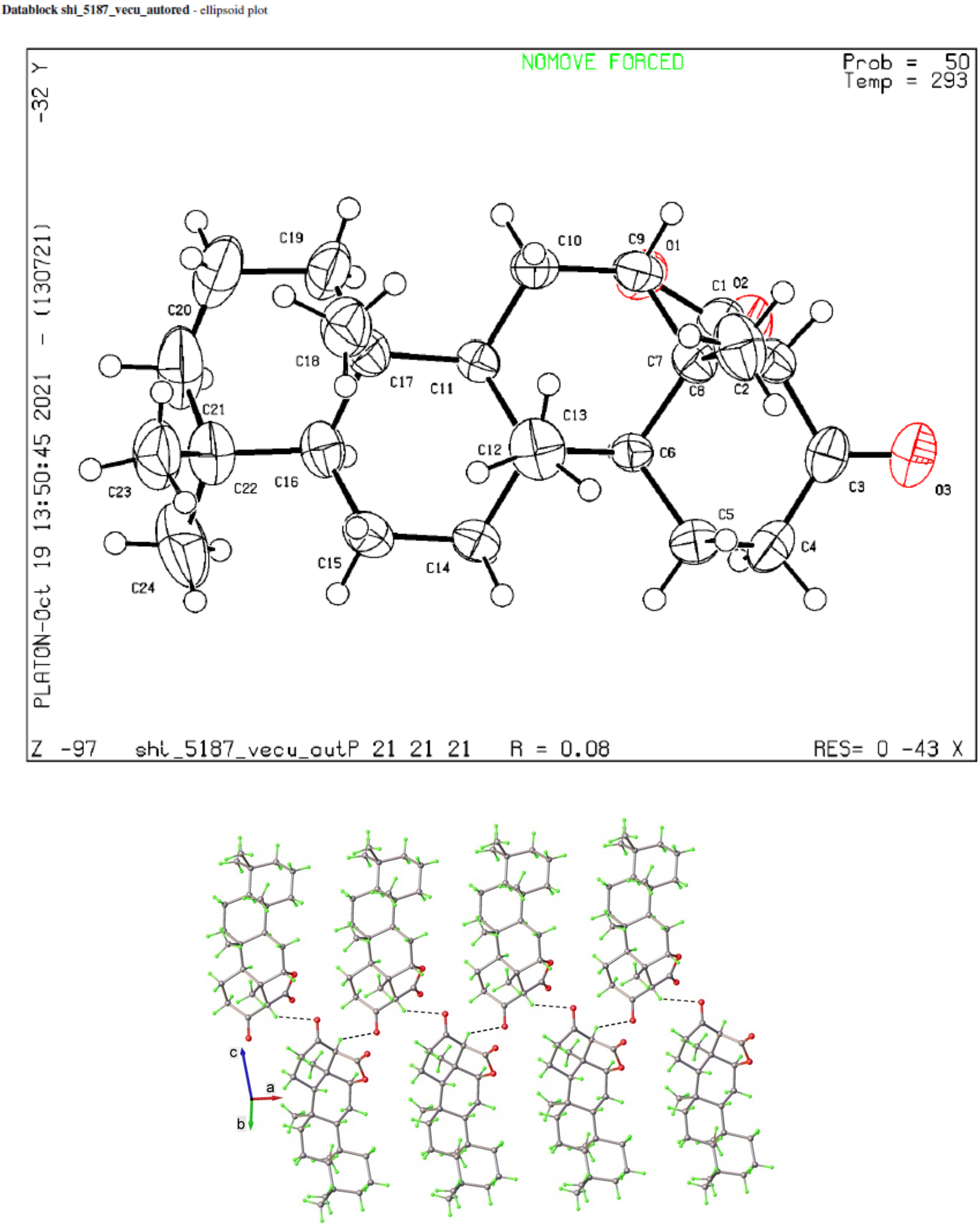

Figure S1. X-ray molecular structure of compound (**8**) and a view of 1D architecture showing the role of intermolecular of H-bonds.

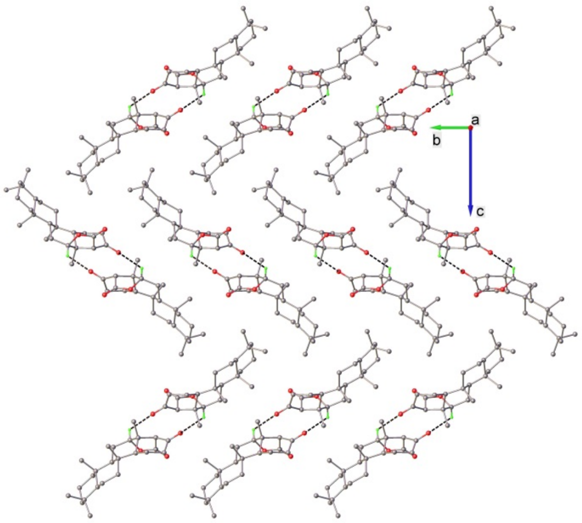

Figure S2. Partial view of the crystal structure along *a* axis.

Table S1. The crystallographic data and refinement details.

	5187
empirical formula	$C_{24}H_{36}O_3$
Fw	372.53
space group	$P2_12_12_1$
a [Å]	7.3866(4)
b [Å]	10.3133(7)
c [Å]	26.874(2)
V [Å^3]	2047.3(2)
Z	4
r_{calcd} [g cm^{-3}]	1.209
Crystal size [mm]	0.30 × 0.02 × 0.02
T [K]	293
μ [mm^{-1}]	0.077

2Θ range [°]	4.23 to 58.912
Reflections collected	9276
Independent reflections	4612[R_{int}=0.0541]
Data/restraints/parameters	4612/0/249
R_1[a]	0.0772
wR_2[b]	0.1231
GOF[c]	1.005
CCDC No.	2116545

[a]$R_1 = \Sigma||F_o| - |F_c||/\Sigma|F_o|$. [b]$wR_2 = \{\Sigma[w(F_o^2-F_c^2)^2]/\Sigma[w(F_o^2)^2]\}^{1/2}$. [c]$GOF = \{\Sigma[w(F_o^2-F_c^2)^2]/(n-p)\}^{1/2}$, where n is the number of reflections and p is the total number of parameters refined [1].

Table S2. (**a**) Bond distances (Å) and (**b**) angles (°) for compound **8**.

**a**		**b**	
O1-C1	1.354(6)	C1-O1-C9	109.6(4)
O1-C9	1.454(6)	O1-C1-C2	109.2(5)
O2-C1	1.195(6)	O2-C1-O1	122.2(5)
O3-C3	1.204(5)	O2-C1-C2	128.5(5)
C1-C2	1.508(6)	C1-C2-C7	102.3(4)
C2-C3	1.501(6)	C3-C2-C1	115.3(4)
C2-C7	1.535(6)	C3-C2-C7	116.4(4)
C3-C4	1.499(7)	O3-C3-C2	120.4(5)
C4-C5	1.524(7)	O3-C3-C4	121.9(5)
C5-C6	1.523(5)	C4-C3-C2	117.6(5)
C6-C7	1.549(6)	C3-C4-C5	112.8(5)

C6-C12	1.547(6)	C6-C5-C4	110.0(4)
C7-C8	1.534(6)	C5-C6-C7	109.5(4)
C7-C9	1.521(6)	C5-C6-C12	119.2(3)
C9-C10	1.505(6)	C12-C6-C7	115.2(4)
C10-C11	1.530(5)	C2-C7-C6	107.3(4)
C11-C12	1.560(5)	C8-C7-C2	109.9(4)
C11-C17	1.549(6)	C8-C7-C6	116.8(4)
C12-C13	1.543(6)	C9-C7-C2	99.8(3)
C12-C14	1.530(6)	C9-C7-C6	109.4(4)
C14-C15	1.518(6)	C9-C7-C8	112.1(4)
C15-C16	1.521(6)	O1-C9-C7	104.3(4)
C16-C17	1.554(6)	O1-C9-C10	110.4(4)
C16-C22	1.546(7)	C10-C9-C7	115.4(4)
C17-C18	1.542(6)	C9-C10-C11	113.8(4)
C17-C19	1.546(6)	C10-C11-C12	110.6(4)
C19-C20	1.525(7)	C10-C11-C17	114.4(4)
C20-C21	1.500(8)	C17-C11-C12	116.8(4)
C21-C22	1.514(7)	C6-C12-C11	105.8(3)
C22-C23	1.525(7)	C13-C12-C6	111.3(4)
C22-C24	1.542(7)	C13-C12-C11	115.9(4)
		C14-C12-C6	108.4(4)
		C14-C12-C11	107.8(4)
		C14-C12-C13	107.4(4)

C15-C14-C12	113.2(4)
C14-C15-C16	111.9(4)
C15-C16-C17	110.4(4)
C15-C16-C22	115.0(4)
C22-C16-C17	117.3(4)
C11-C17-C16	106.3(4)
C18-C17-C11	111.7(4)
C18-C17-C16	114.3(4)
C18-C17-C19	108.2(4)
C19-C17-C11	108.4(4)
C19-C17-C16	107.6(4)
C20-C19-C17	112.4(4)
C21-C20-C19	111.9(5)
C20-C21-C22	114.4(5)
C21-C22-C16	107.5(5)
C21-C22-C23	110.4(5)
C21-C22-C24	107.2(6)
C23-C22-C16	115.1(5)
C23-C22-C24	107.3(5)
C24-C22-C16	109.0(5)

NMR SPECTRA

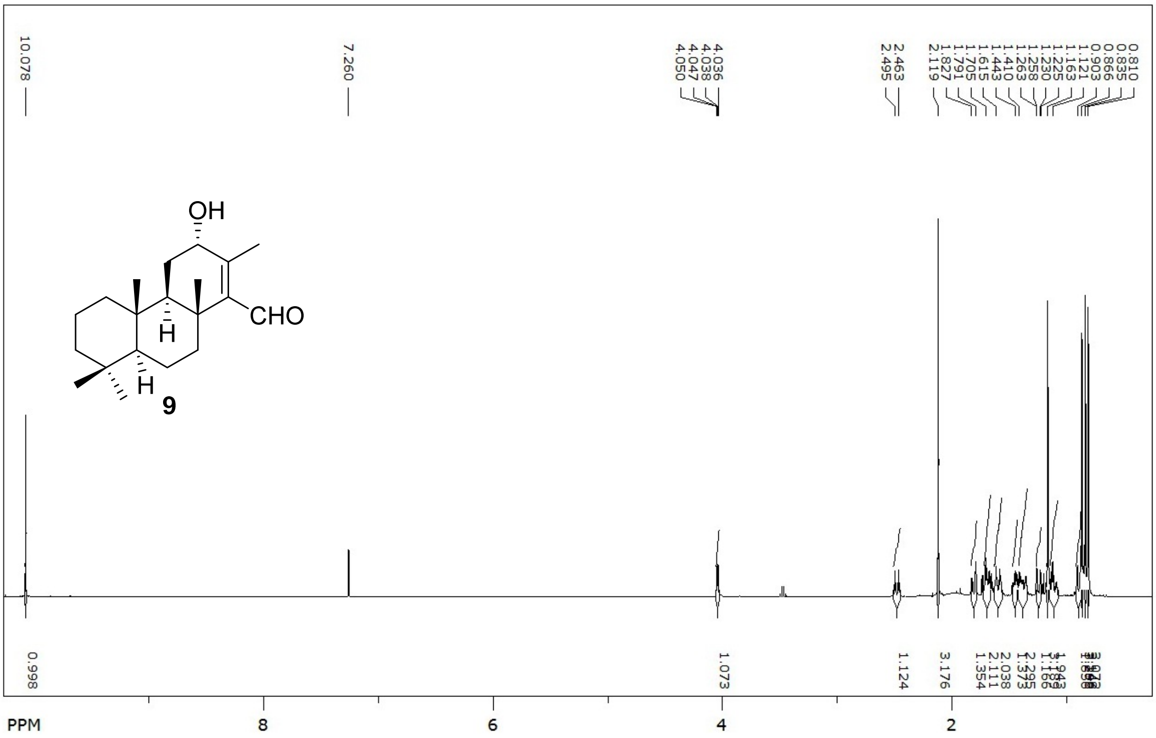

^{1}H-NMR spectrum for compound **9** ($CDCl_3$, 400.13 MHz).

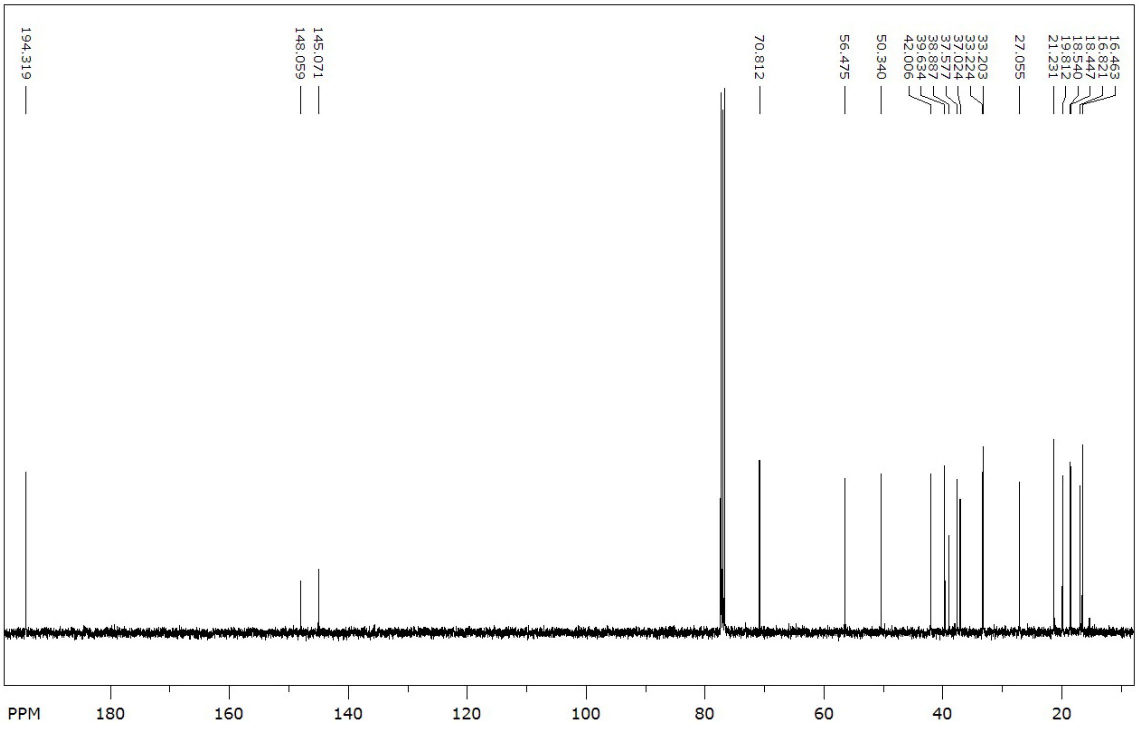

^{13}C-NMR spectrum for compound **9** ($CDCl_3$, 100.61 MHz).

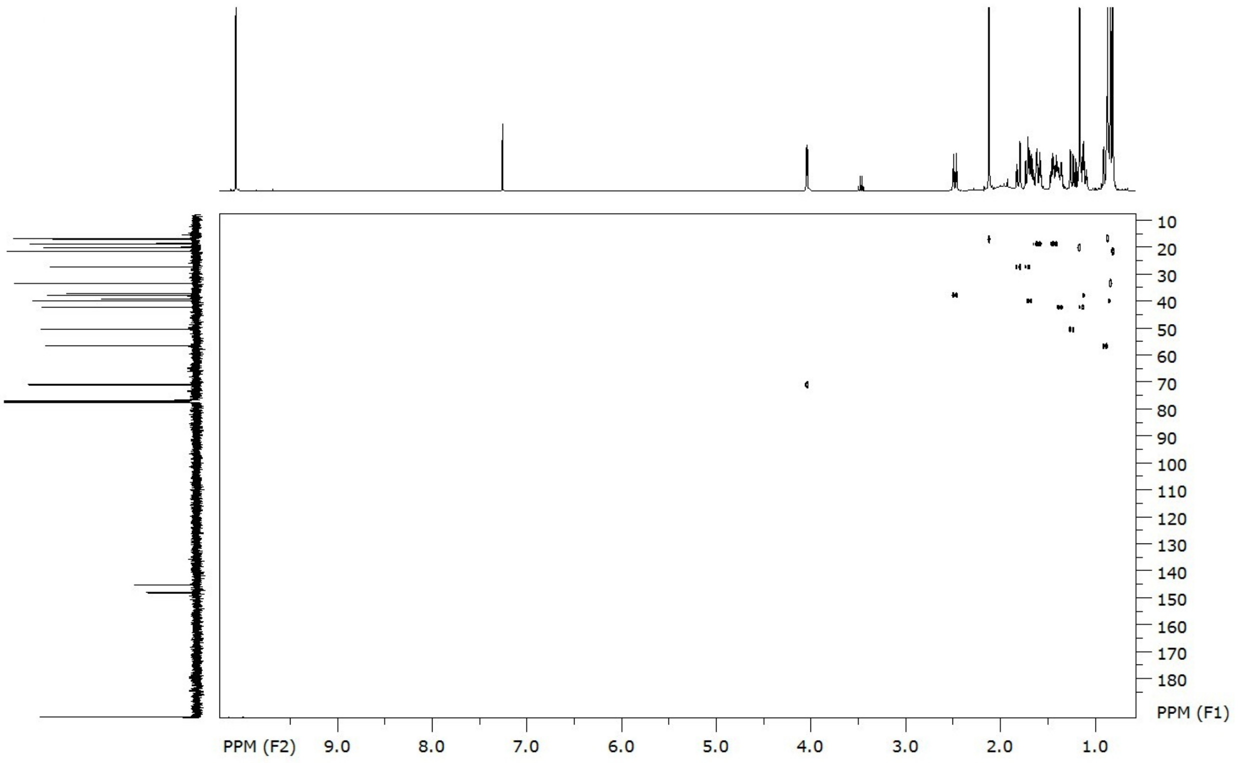

^{1}H, ^{13}C HSQC spectrum for compound **9**.

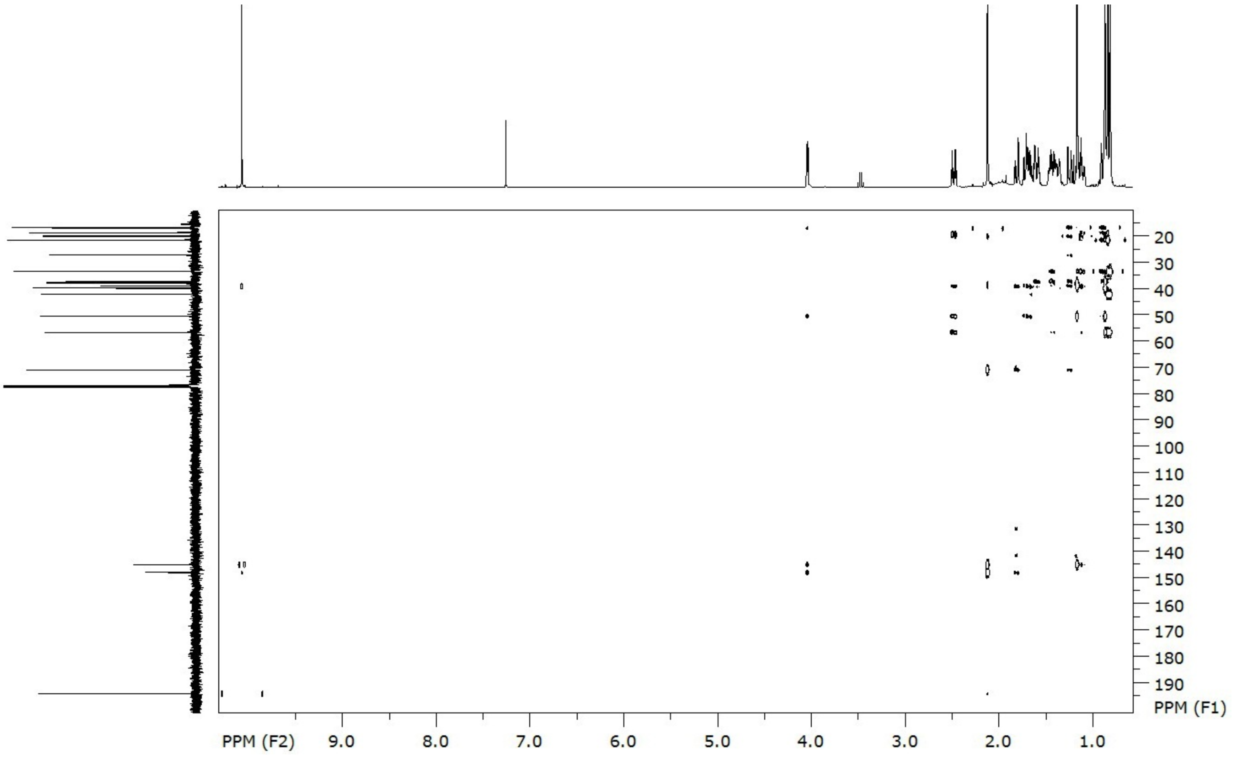

^{1}H, ^{13}C HMBC spectrum for compound **9**.

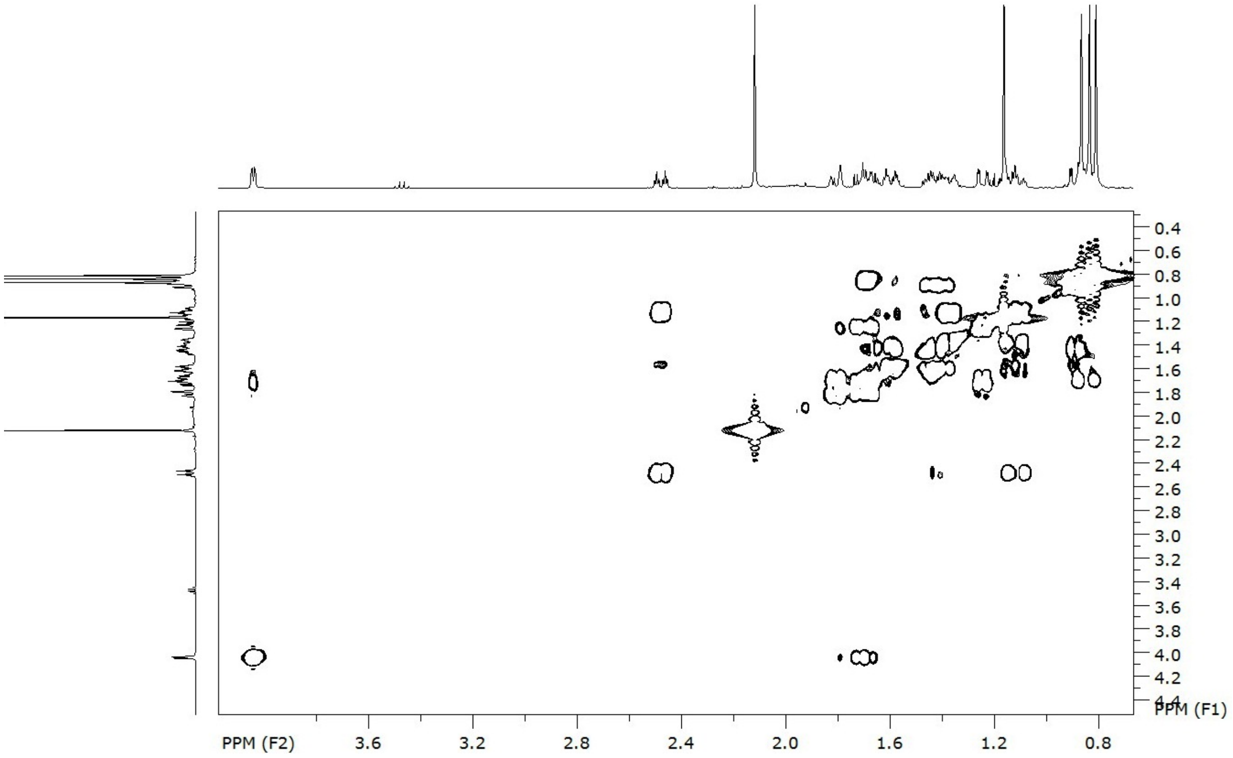

^{1}H-^{1}H COSY spectrum for compound **9**.

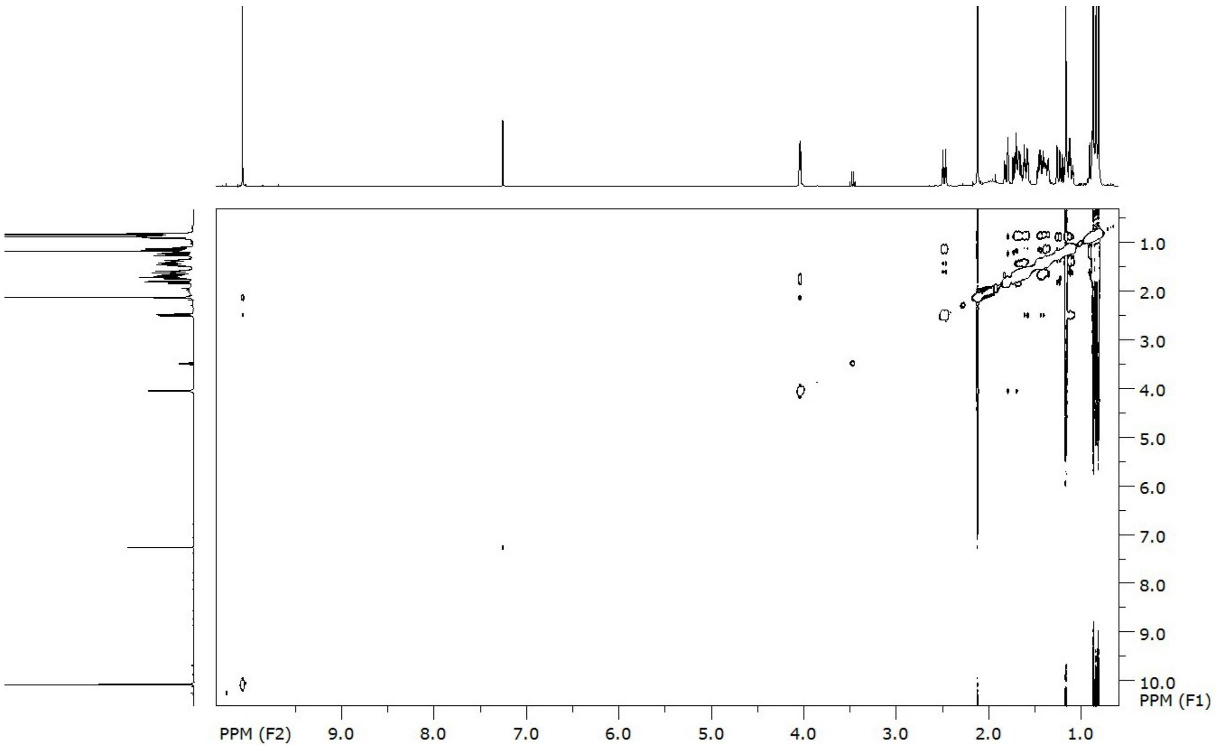

^{1}H-^{1}H NOESY spectrum for compound **9**.

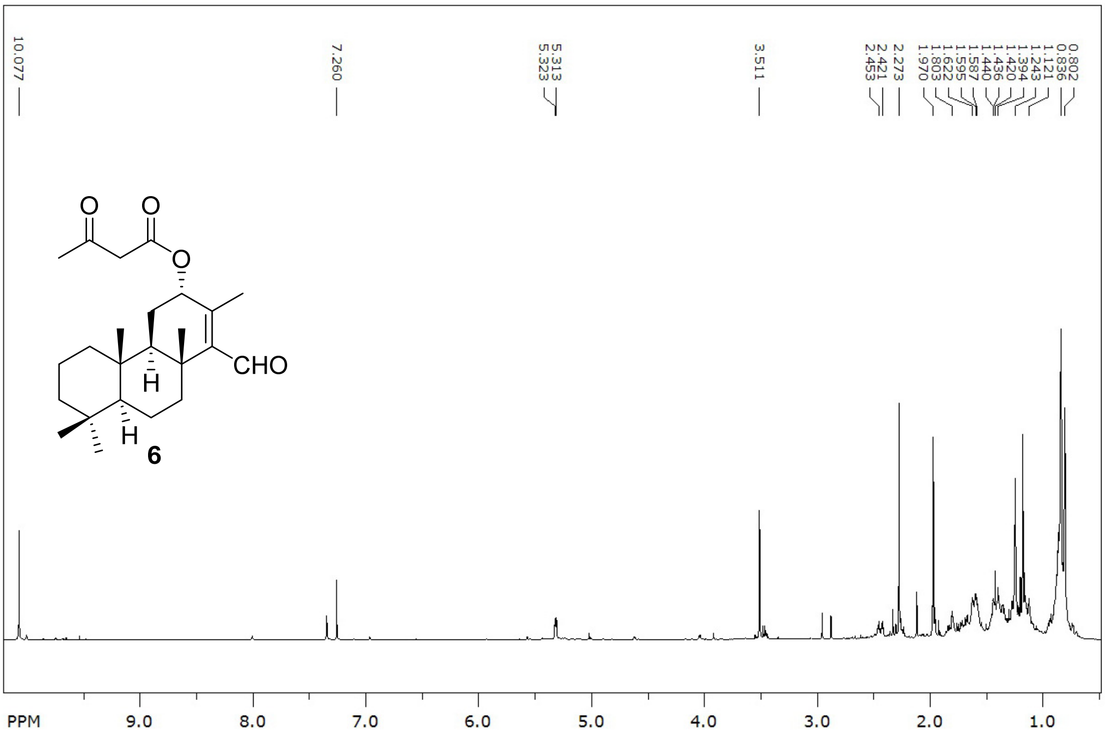

^{1}H-NMR spectrum for compound **6** ($CDCl_3$, 400.13 MHz).

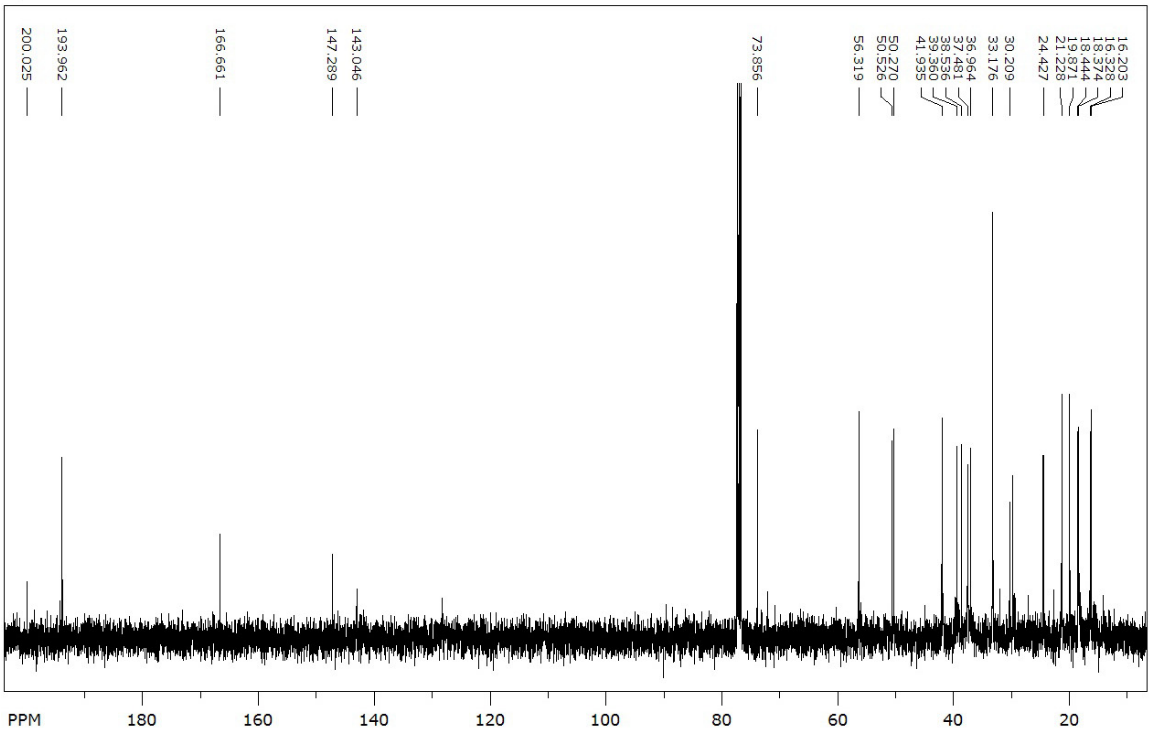

^{13}C-NMR spectrum for compound **6** ($CDCl_3$, 100.61 MHz).

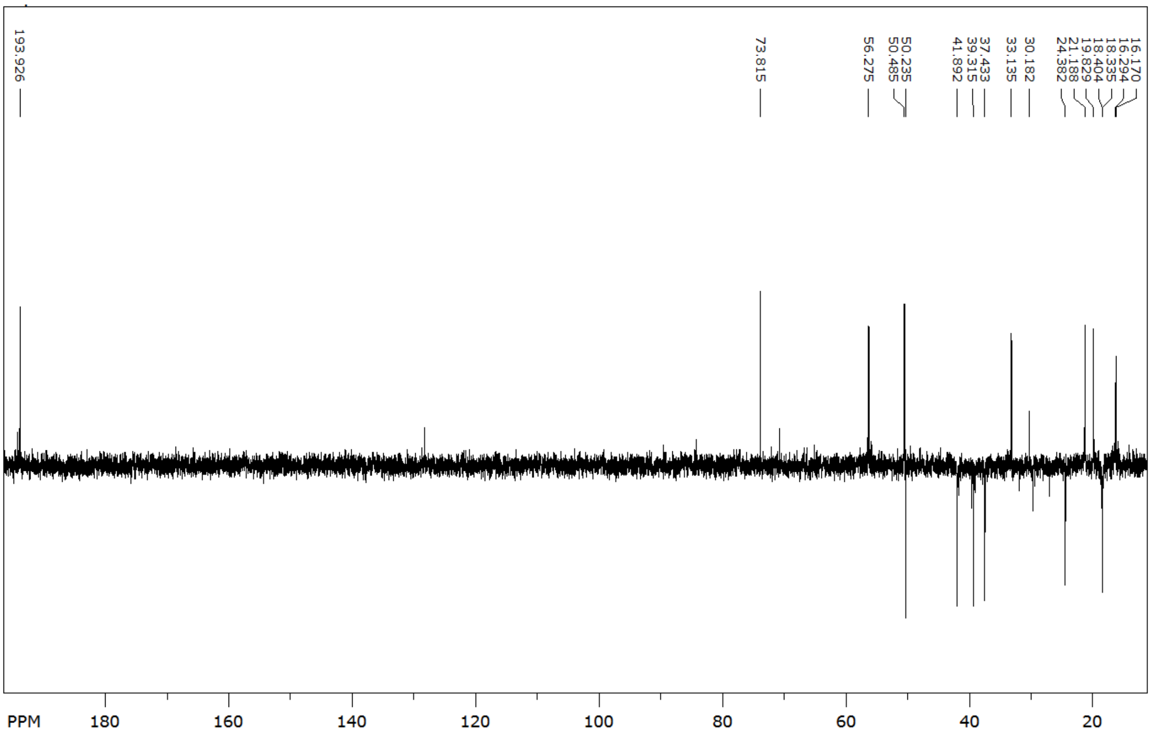

^{13}C DEPT spectrum for compound **6** ($CDCl_3$, 100.61 MHz).

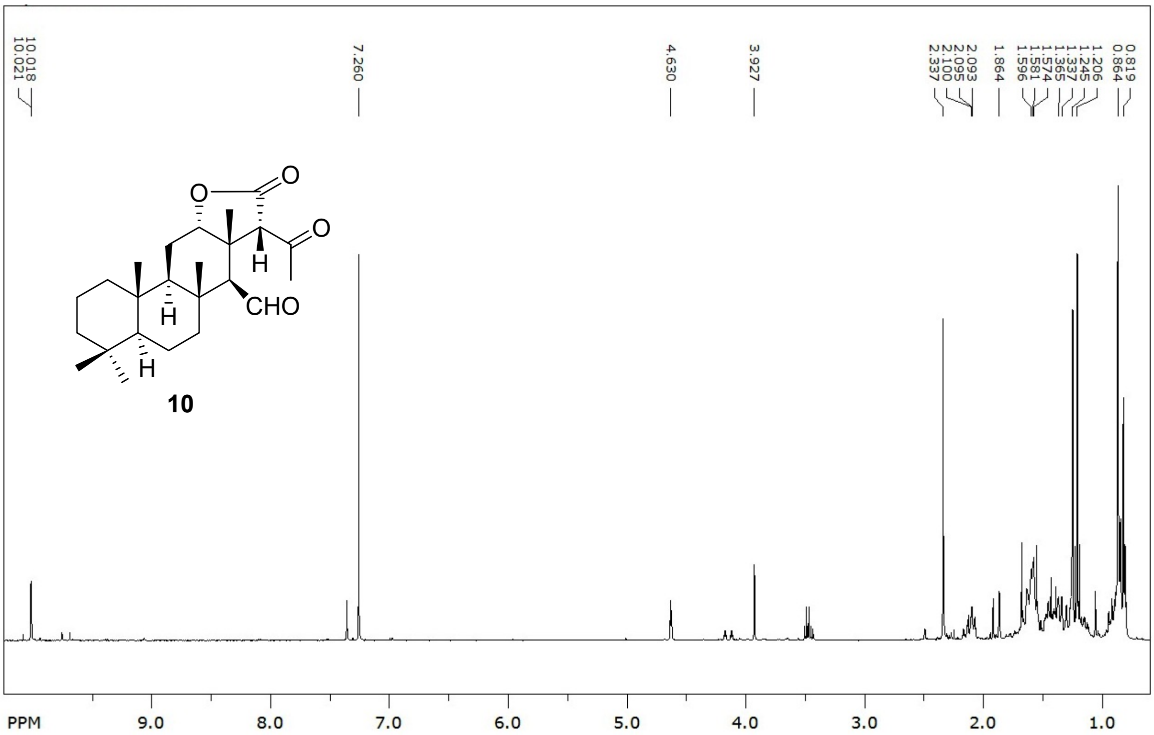

^{1}H-NMR spectrum for compound **10** ($CDCl_3$, 400.13 MHz).

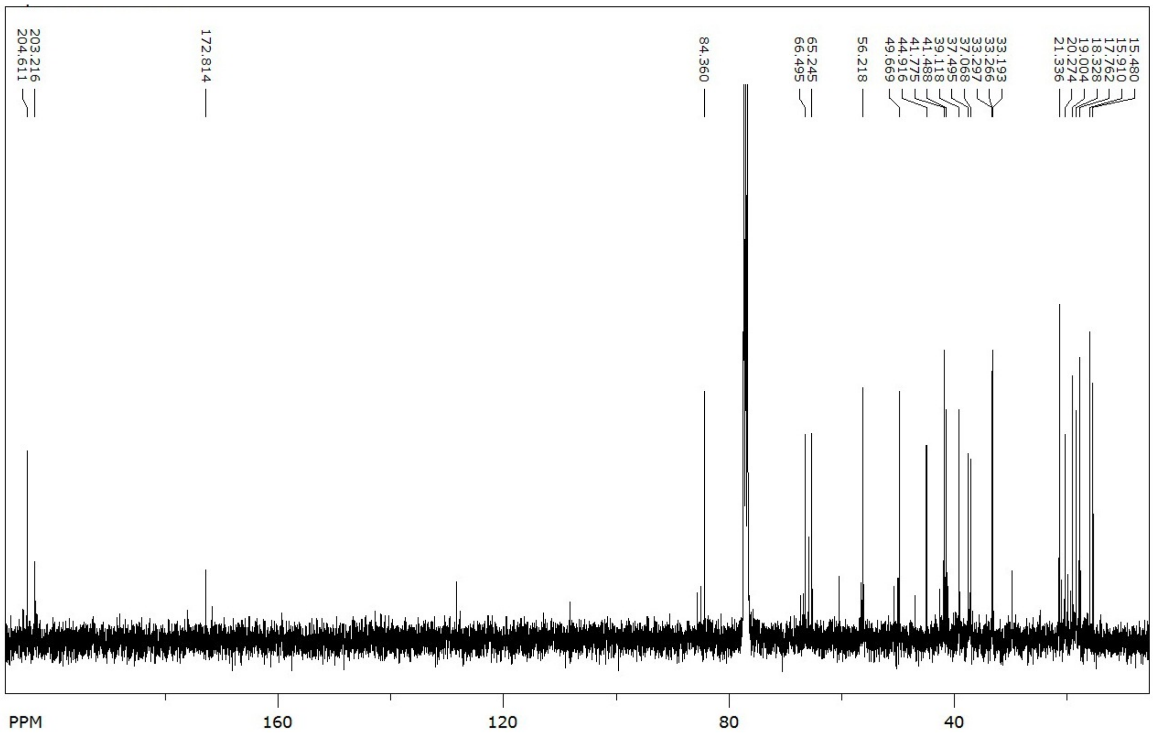

^{13}C-NMR spectrum for compound **10** ($CDCl_3$, 100.61 MHz).

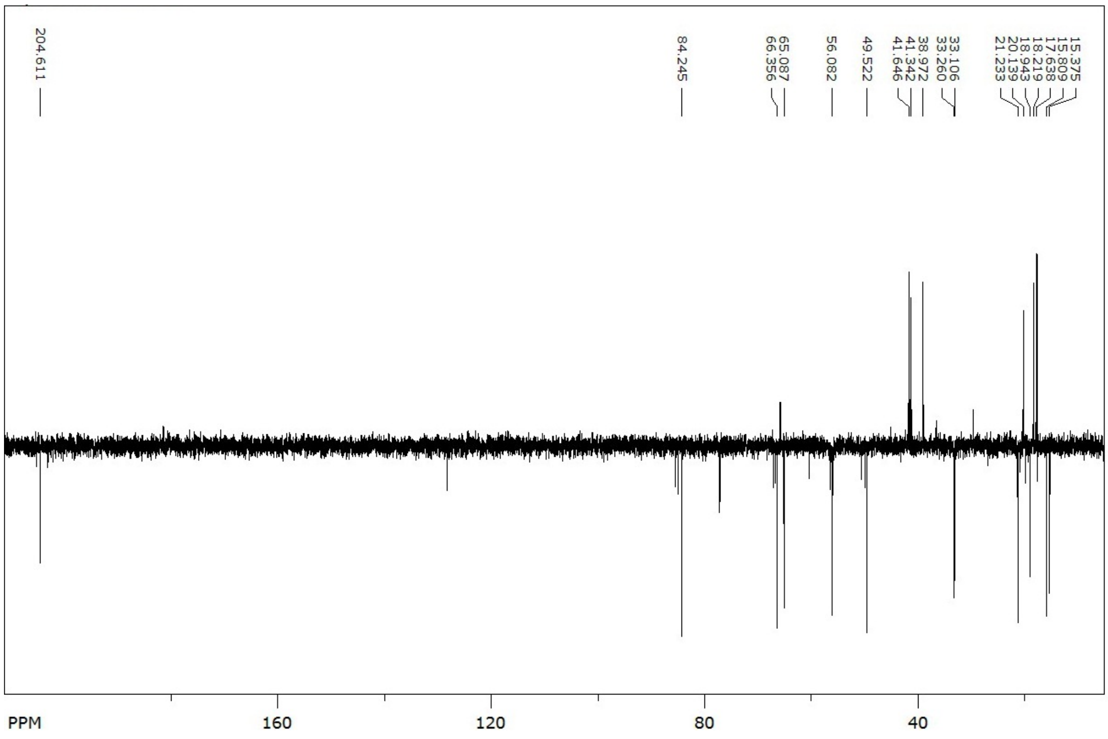

^{13}C DEPT spectrum for compound **10** ($CDCl_3$, 100.61 MHz).

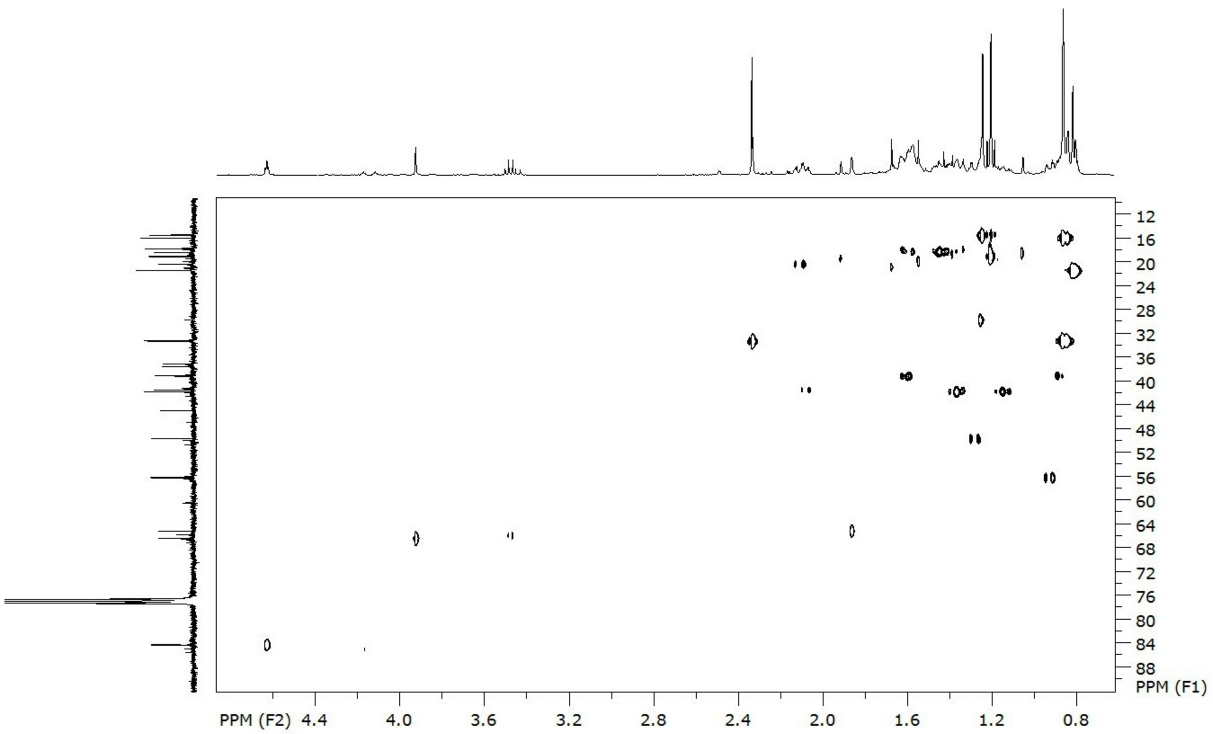

^{1}H, ^{13}C HSQC spectrum for compound **10**.

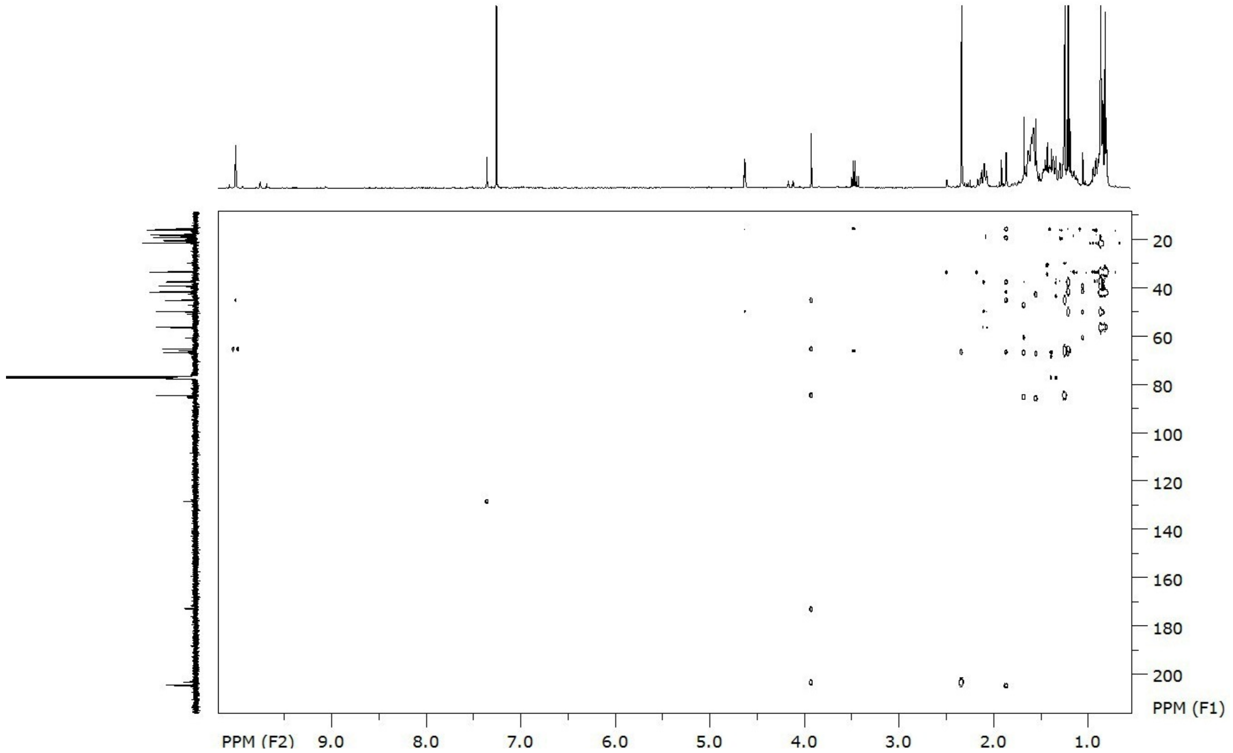

^{1}H, ^{13}C HMBC spectrum for compound **10**.

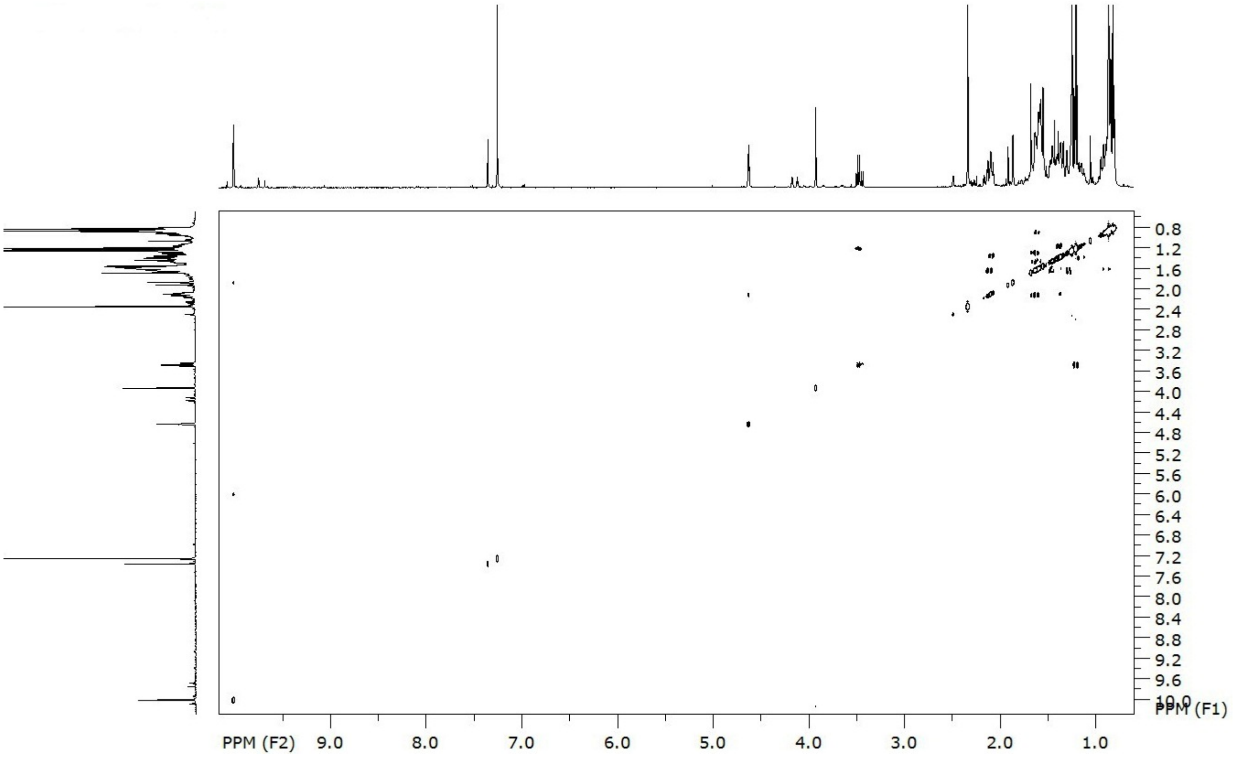

^{1}H-^{1}H COSY spectrum for compound **10**.

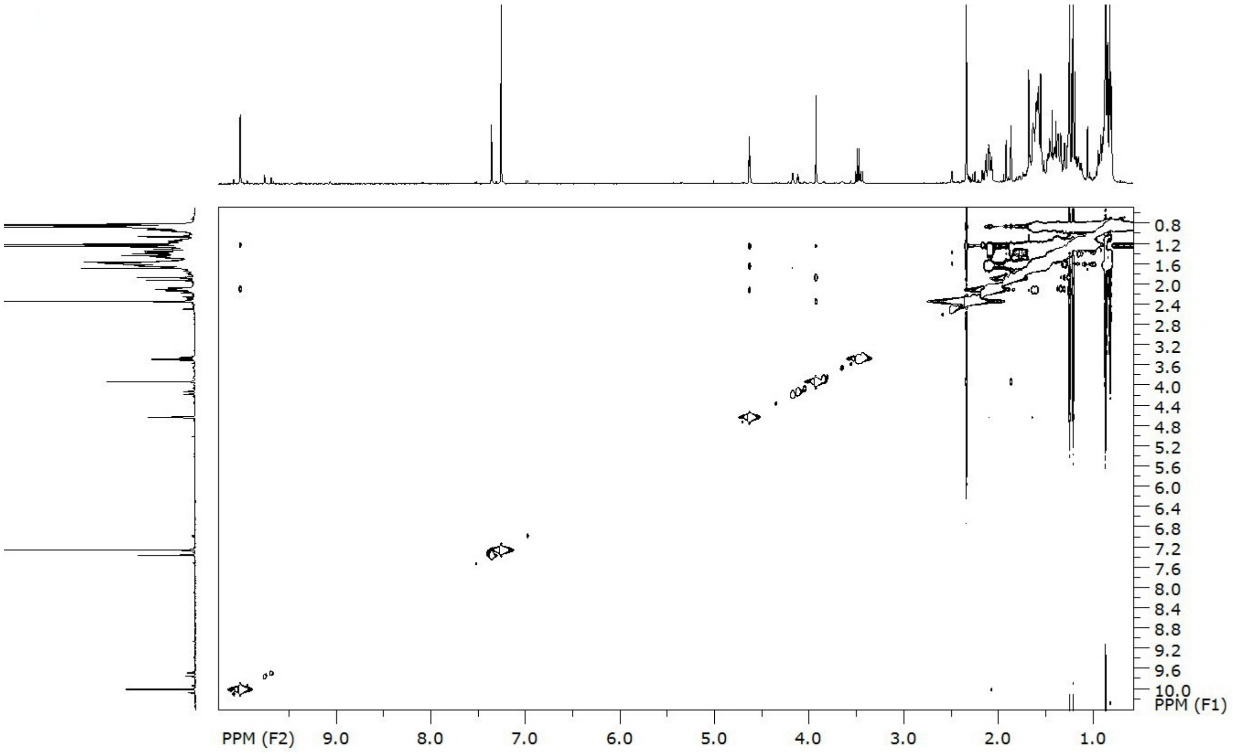

^{1}H-^{1}H NOESY spectrum for compound **10.**

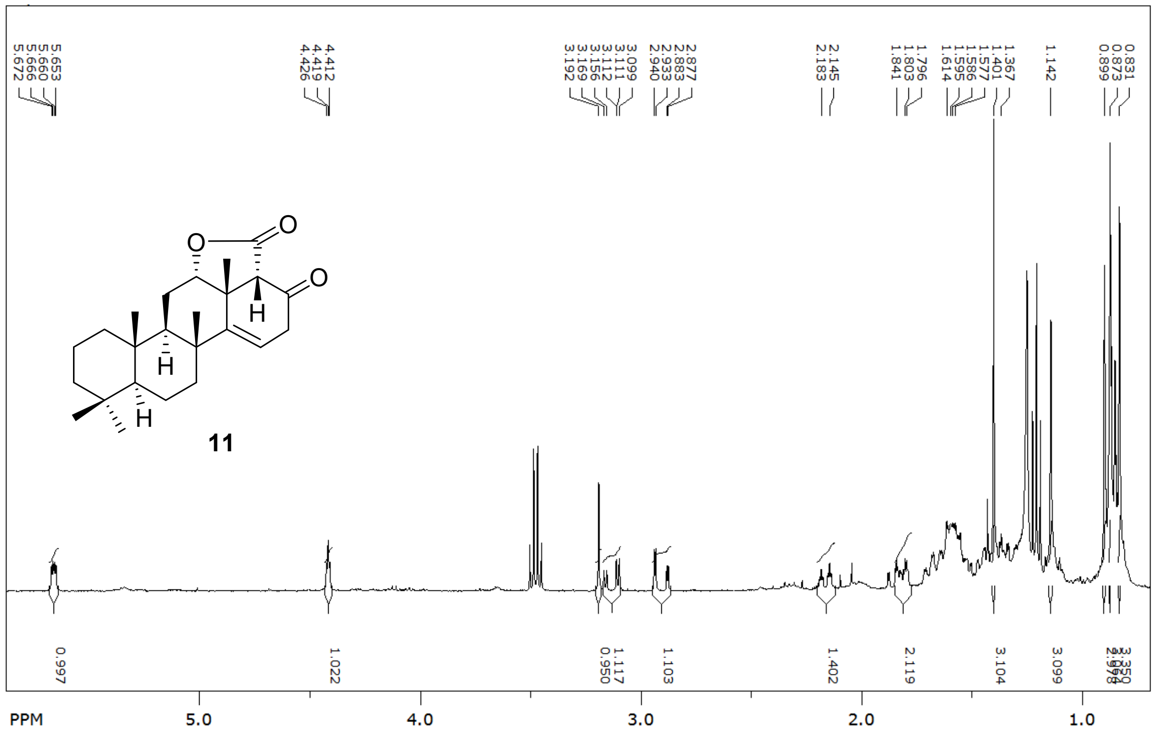

^{1}H-NMR spectrum for compound **11** ($CDCl_3$, 400.13 MHz).

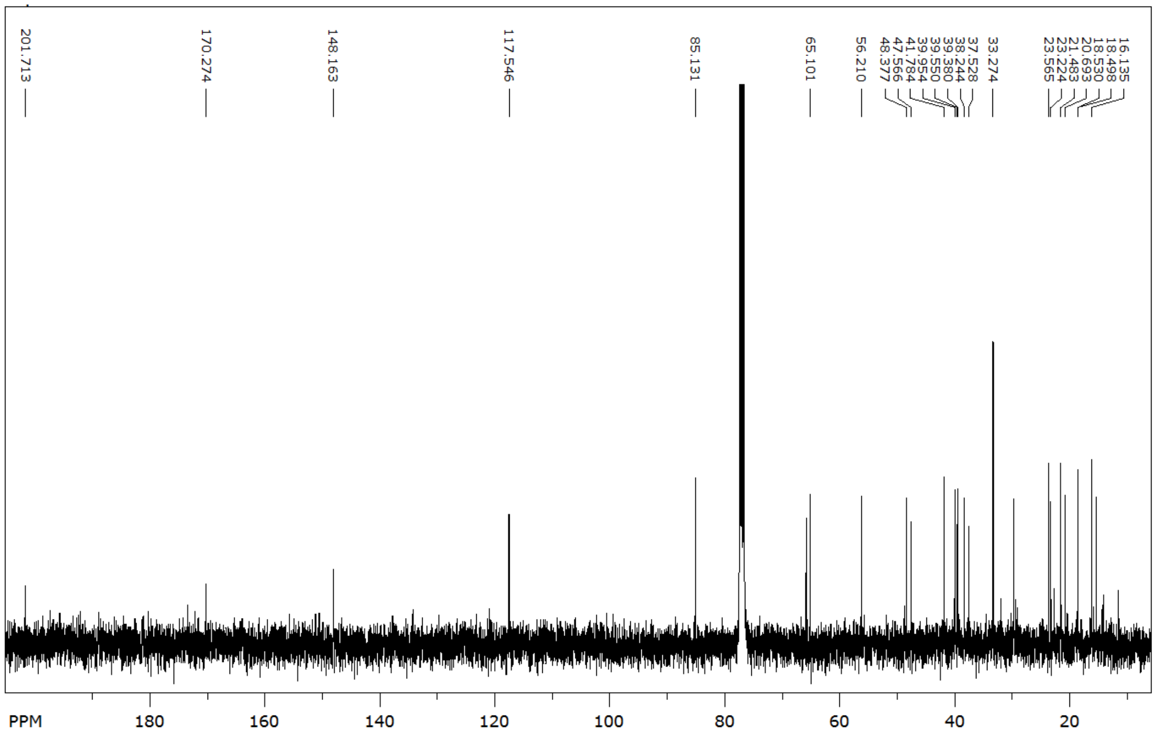

^{13}C-NMR spectrum for compound **11** ($CDCl_3$, 100.61 MHz).

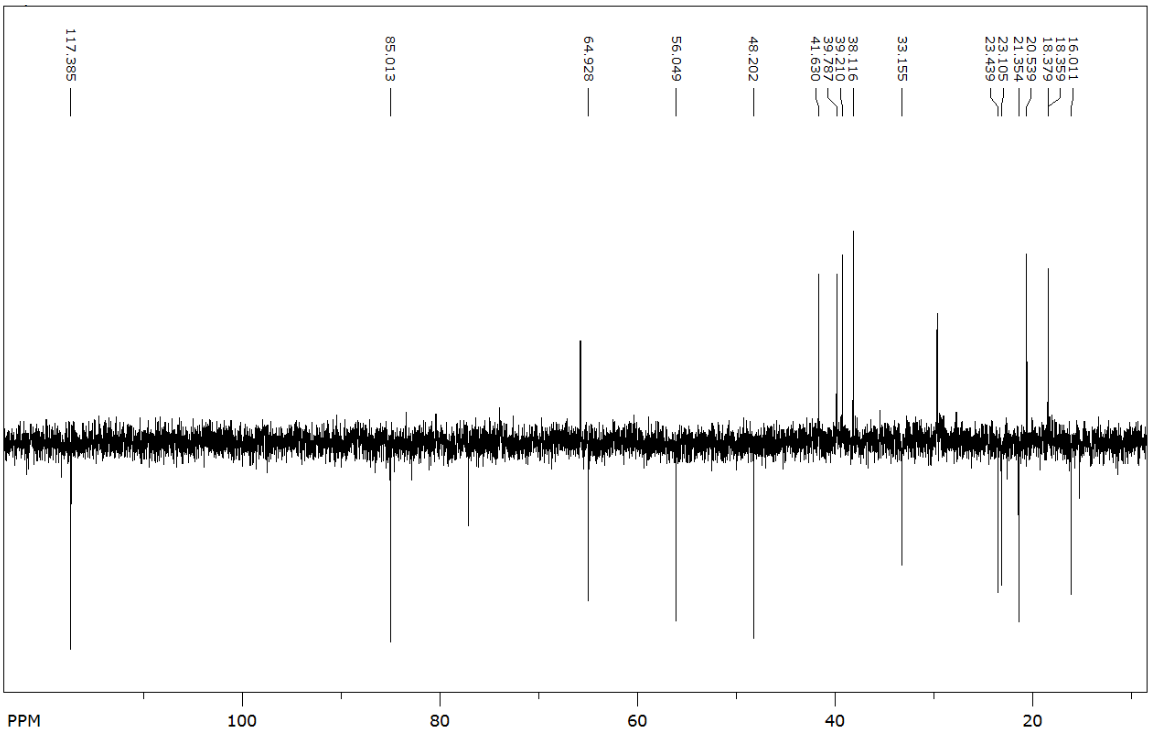

^{13}C DEPT spectrum for compound **11** ($CDCl_3$, 100.61 MHz).

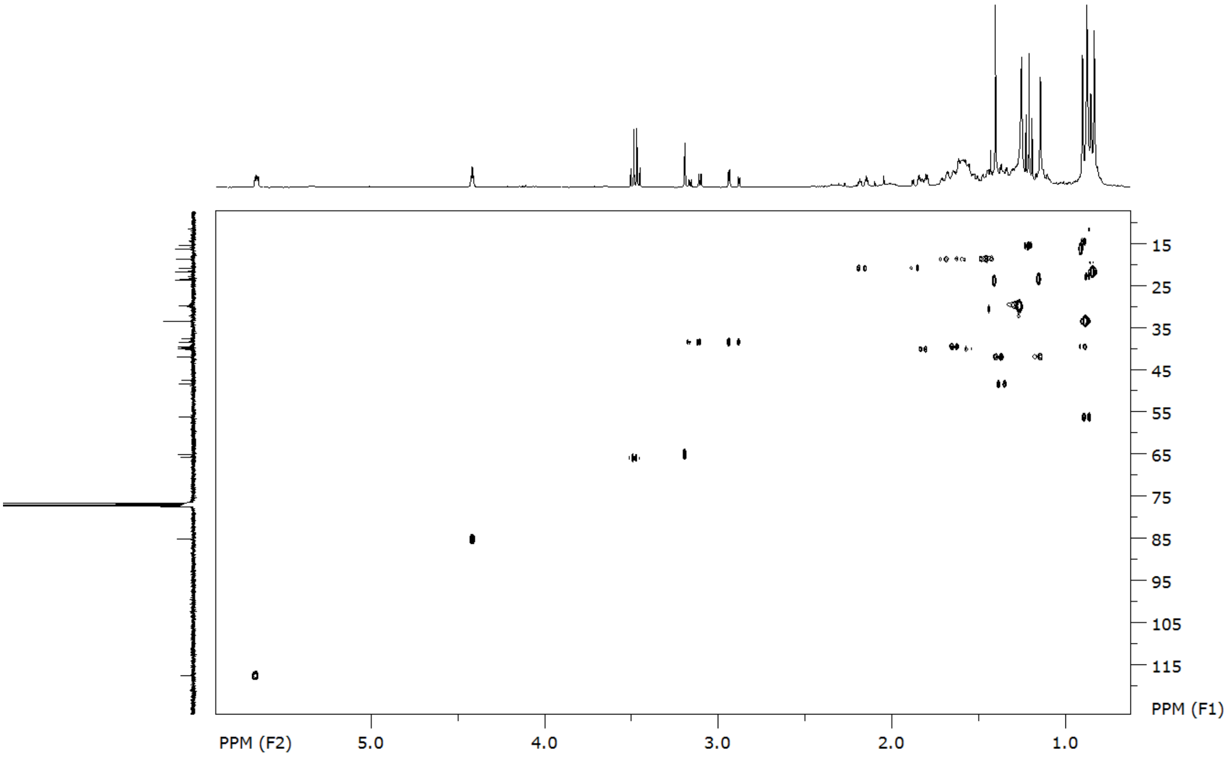

^{1}H, ^{13}C HSQC spectrum for compound **11**.

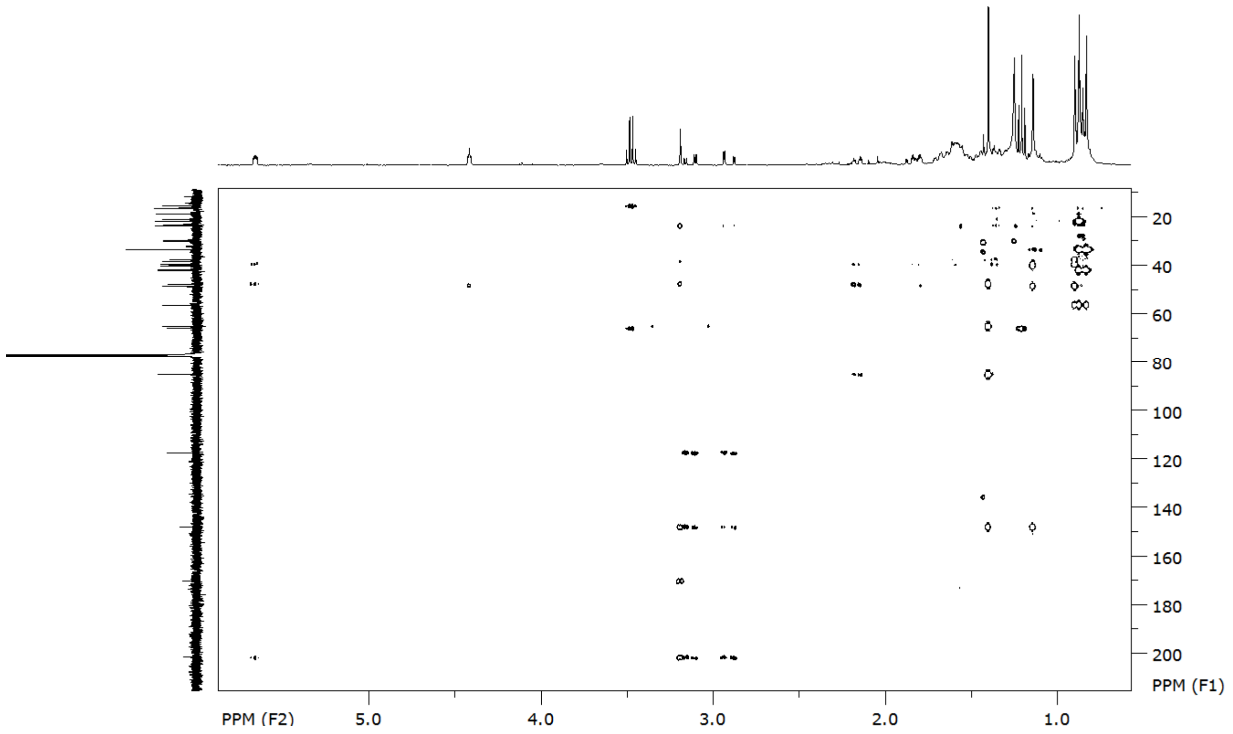

^{1}H, ^{13}C HMBC spectrum for compound **11**.

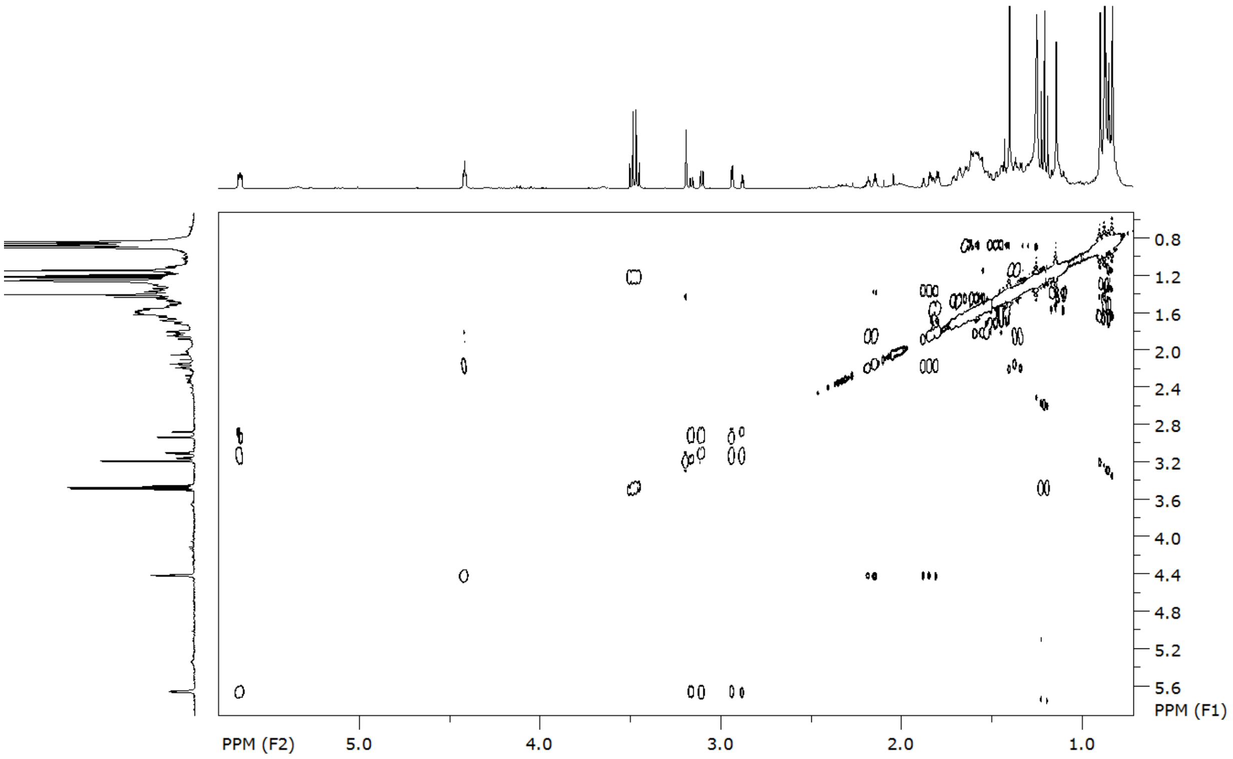

^{1}H-^{1}H COSY spectrum for compound **11**.

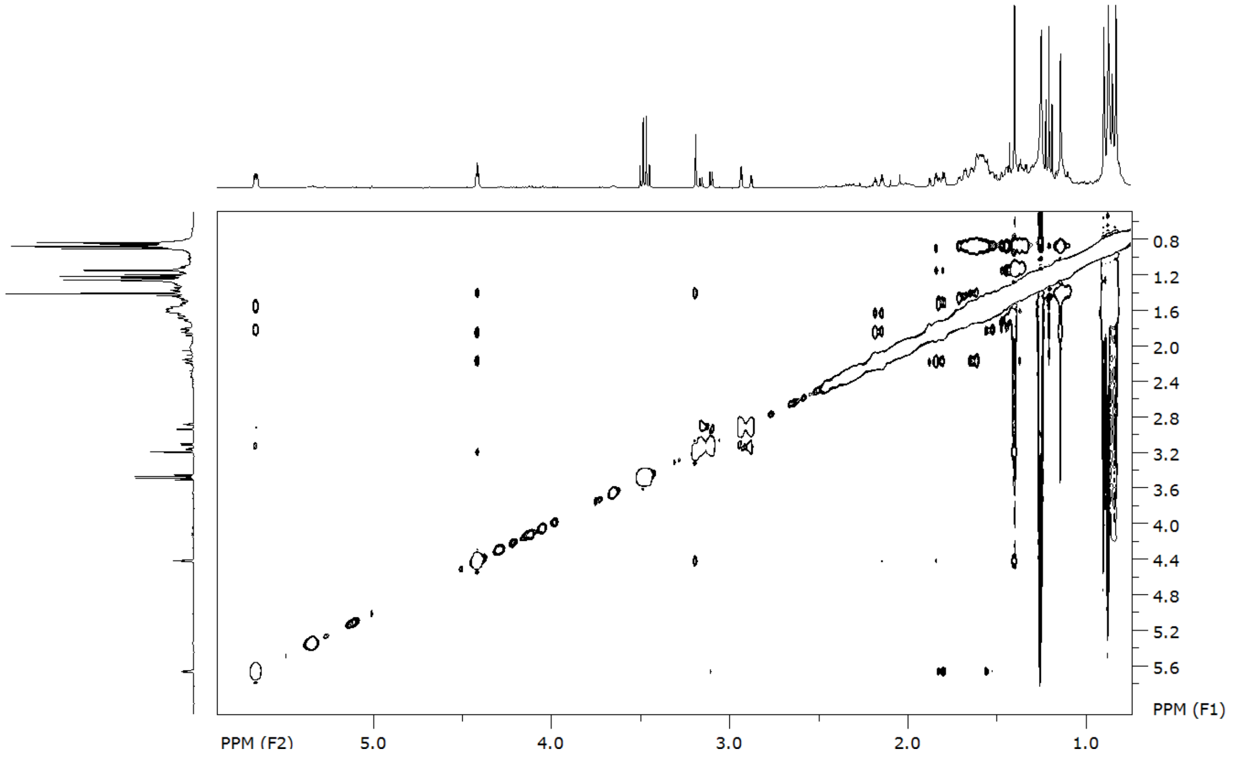

^{1}H-^{1}H NOESY spectrum for compound **11**.

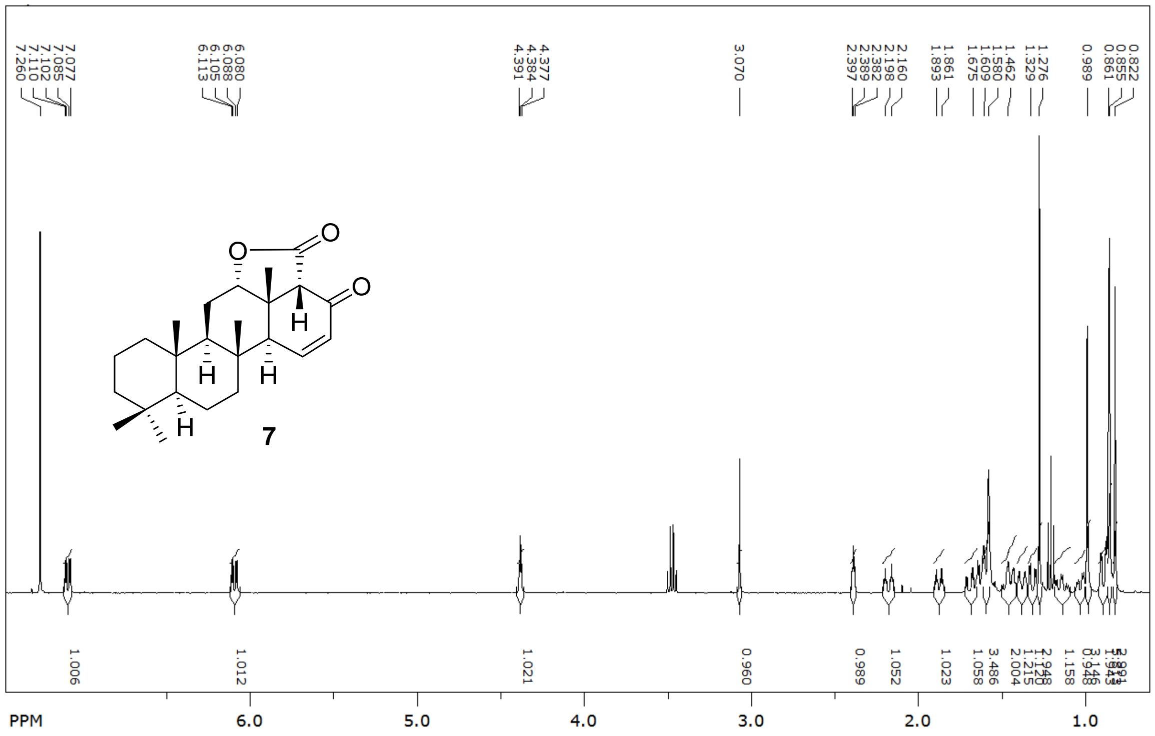

^{1}H-NMR spectrum for compound **7** ($CDCl_3$, 400.13 MHz).

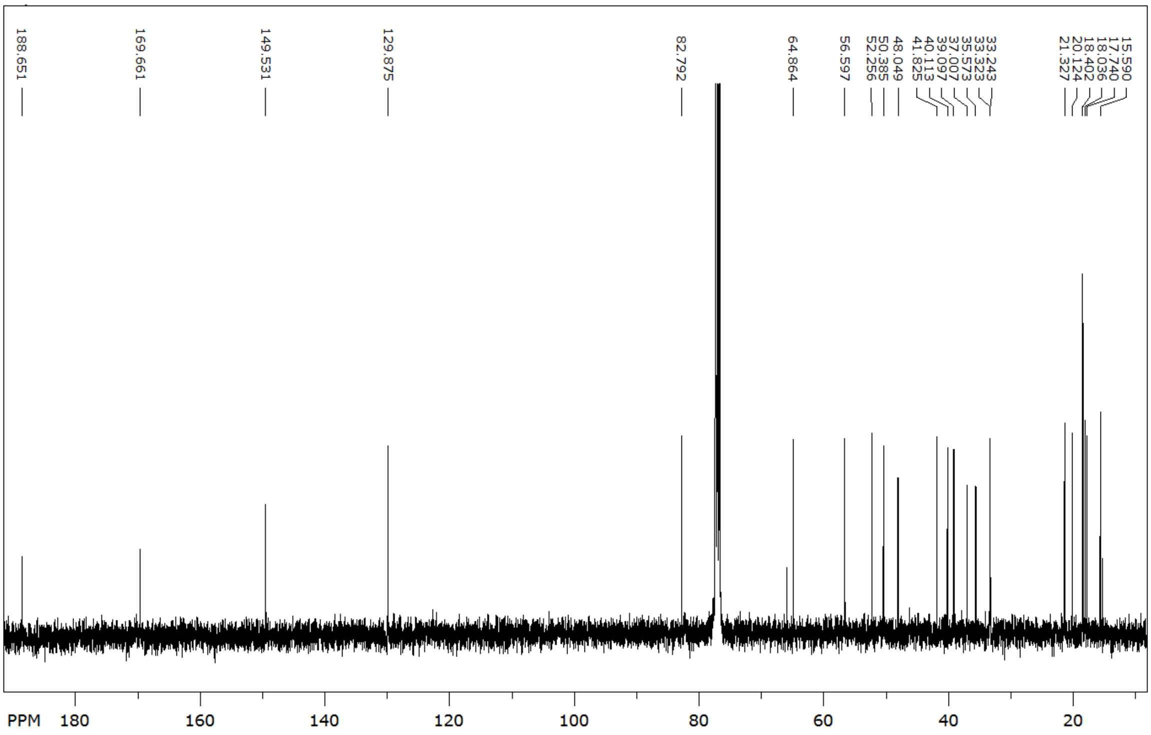

^{13}C-NMR spectrum for compound **7** ($CDCl_3$, 100.61 MHz).

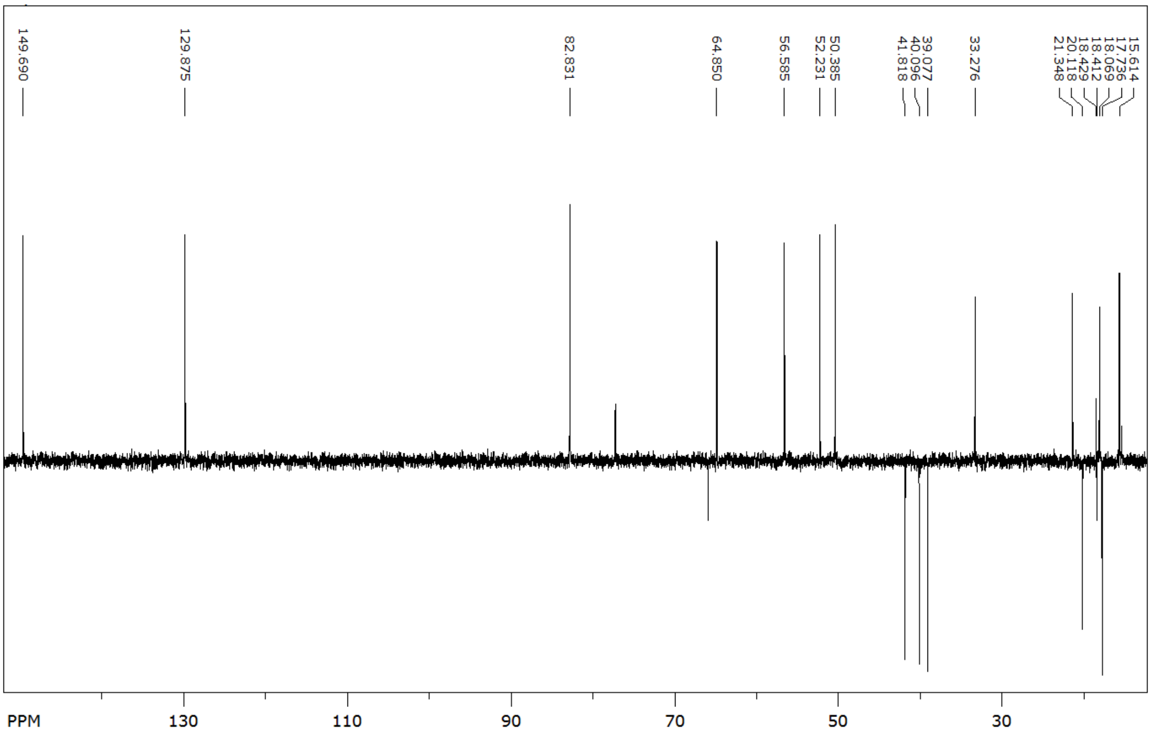

^{13}C DEPT spectrum for compound **7** ($CDCl_3$, 100.61 MHz).

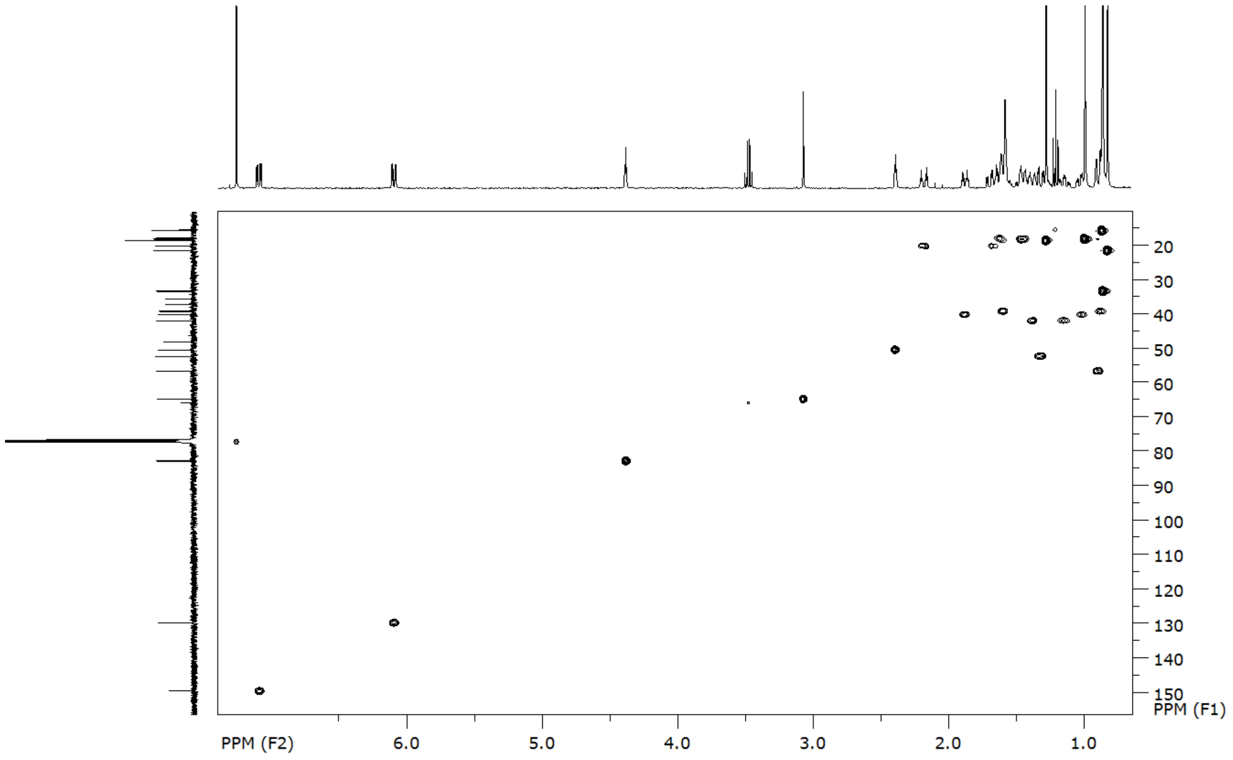

^1H, ^{13}C HSQC spectrum for compound **7**.

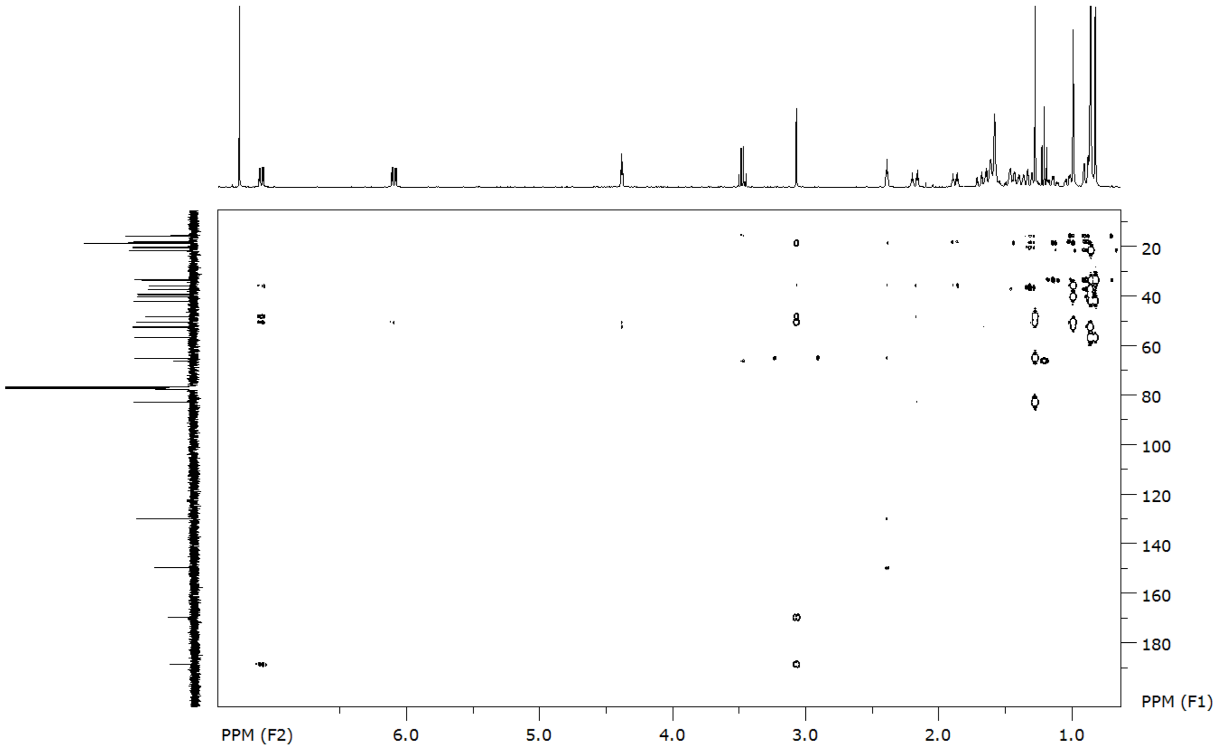

^1H, ^{13}C HMBC spectrum for compound **7**.

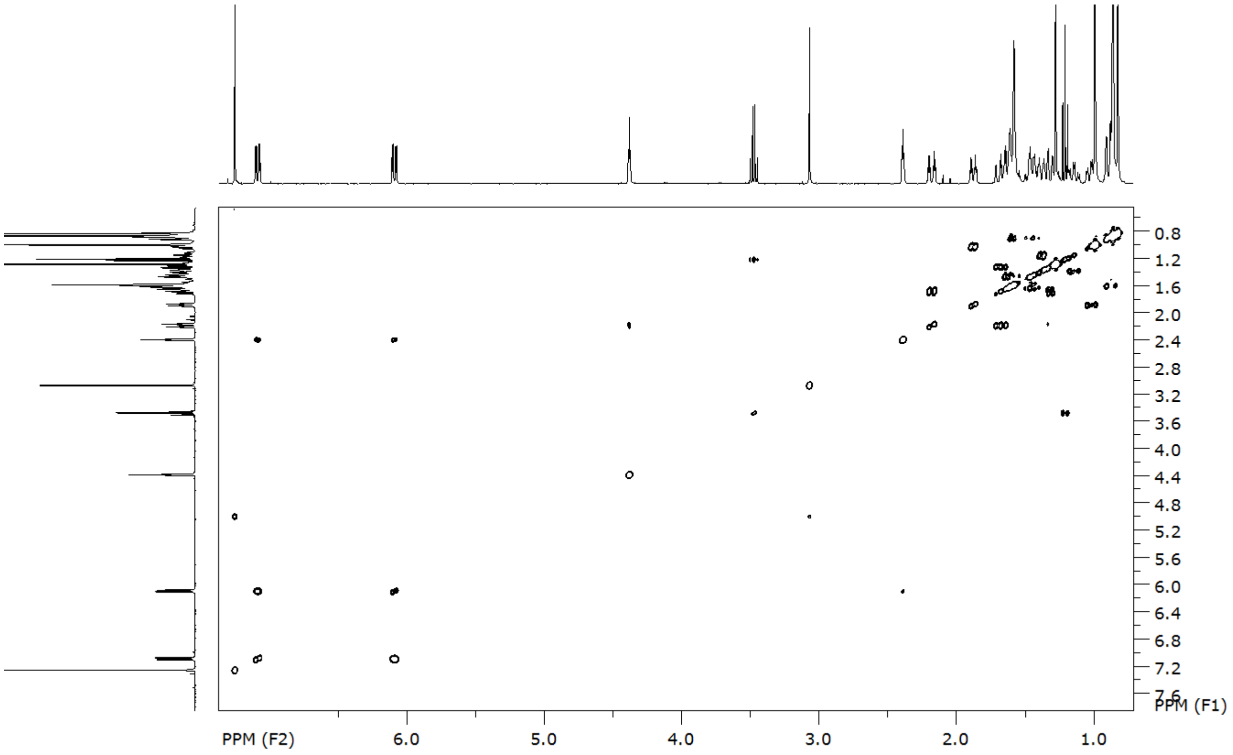

^{1}H-^{1}H COSY spectrum for compound **7**.

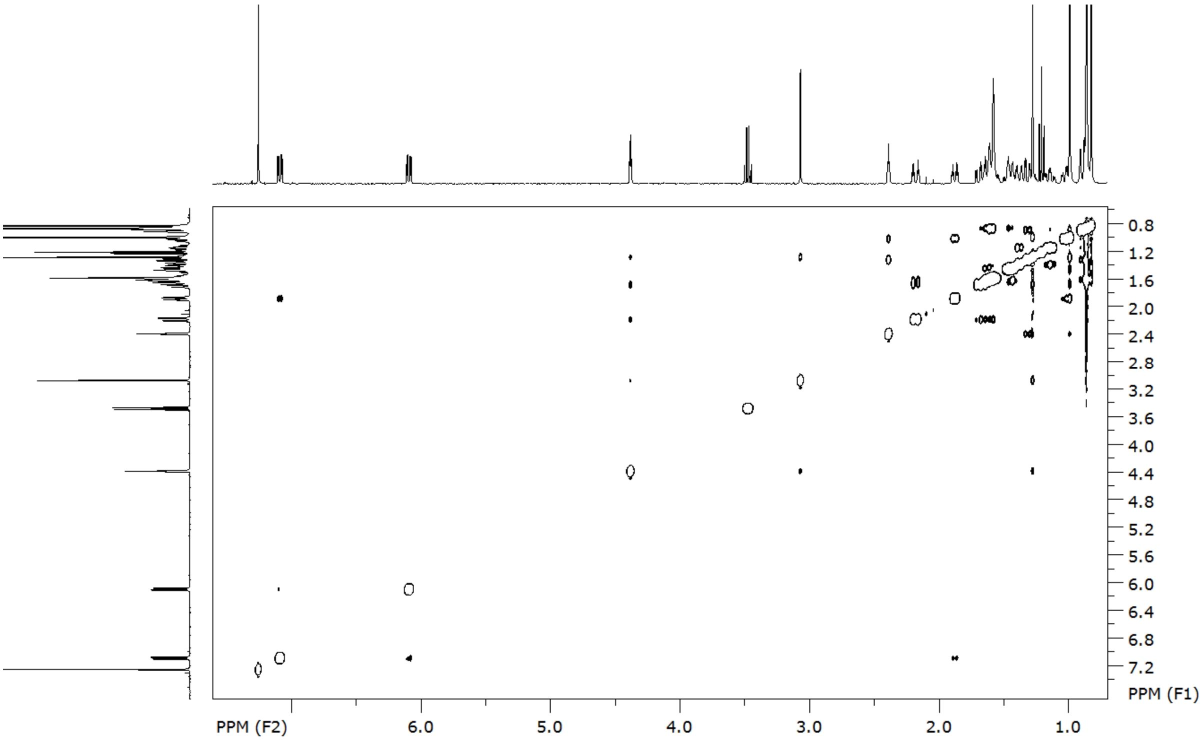

^{1}H-^{1}H NOESY spectrum for compound **7**.

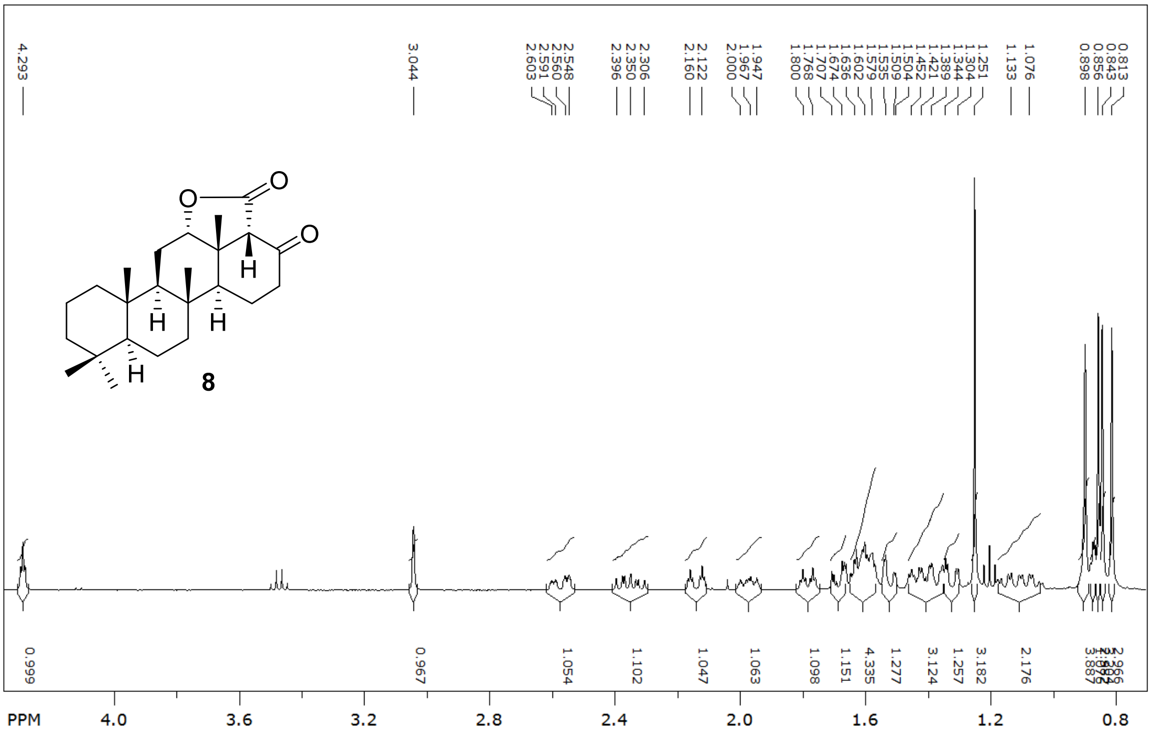

^{1}H-NMR spectrum for compound **8** ($CDCl_3$, 400.13 MHz).

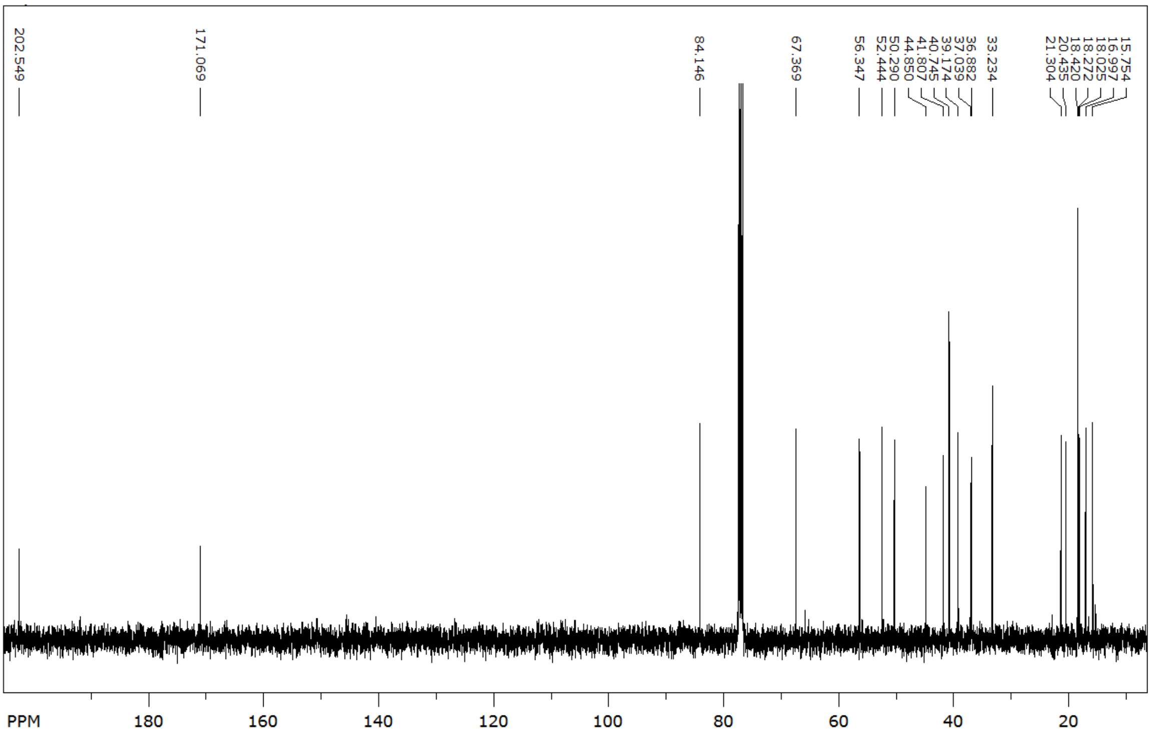

^{13}C-NMR spectrum for compound **8** ($CDCl_3$, 100.61 MHz).

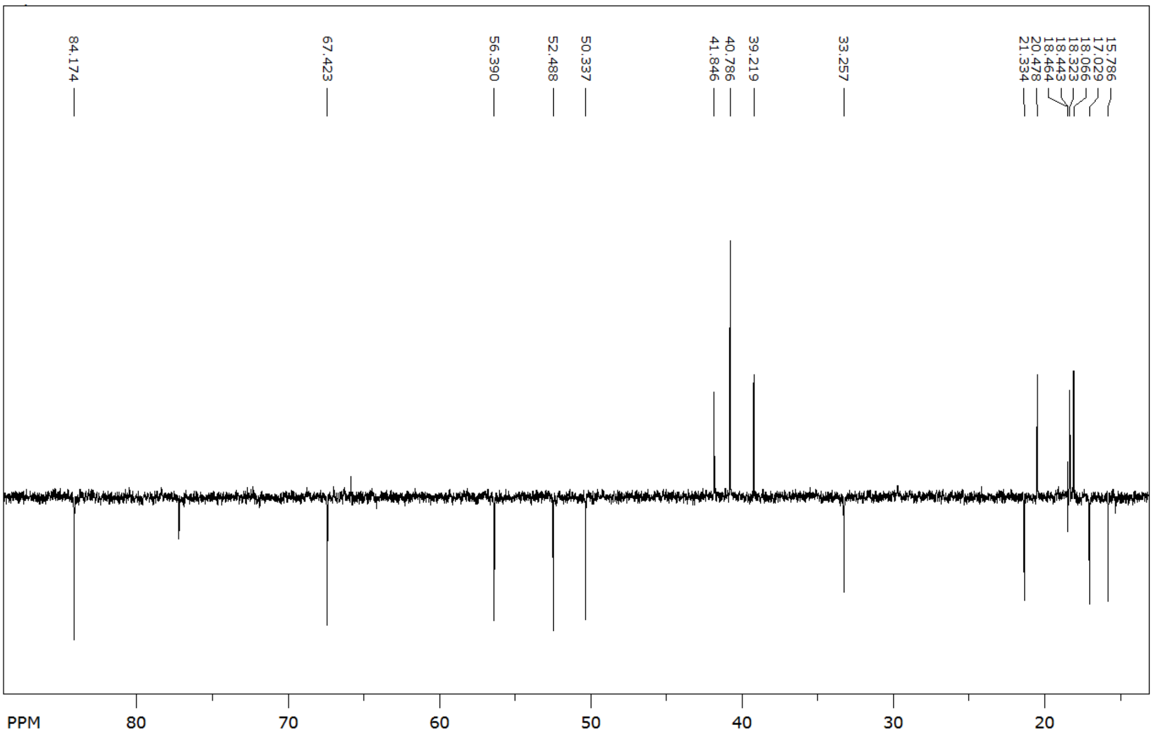

^{13}C DEPT spectrum for compound **8** ($CDCl_3$, 100.61 MHz).

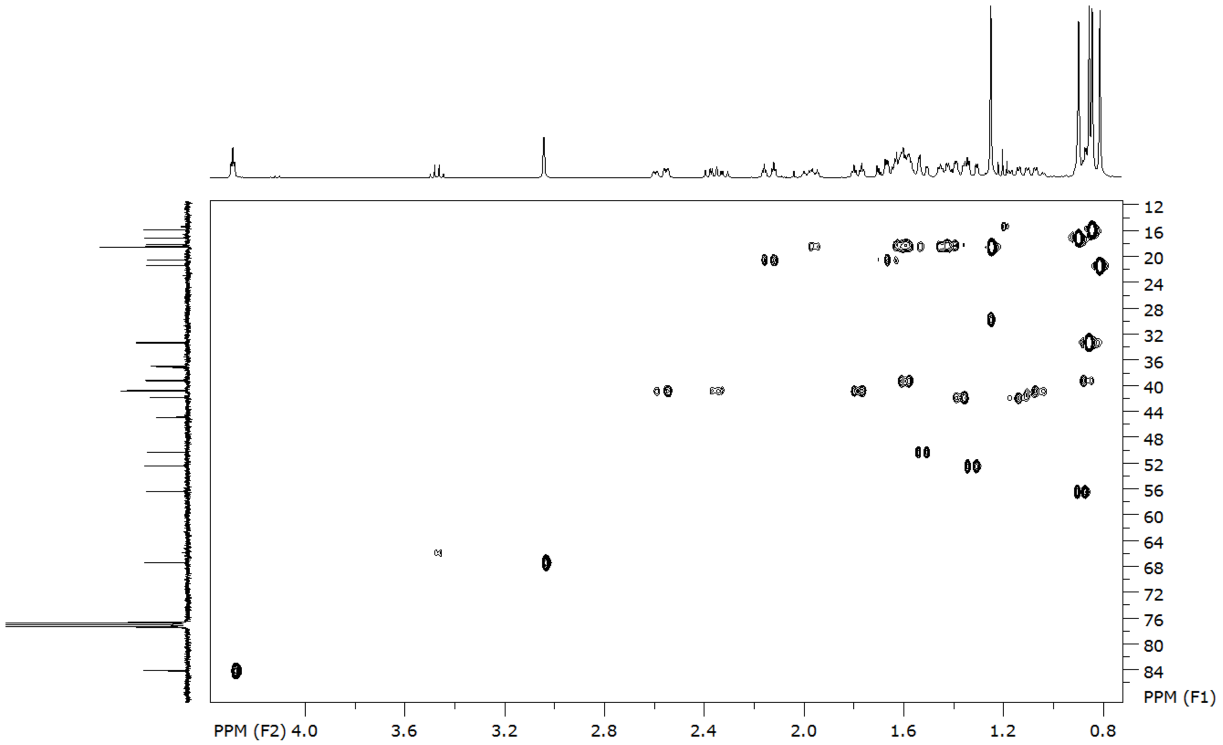

^{1}H, ^{13}C HSQC spectrum for compound **8**.

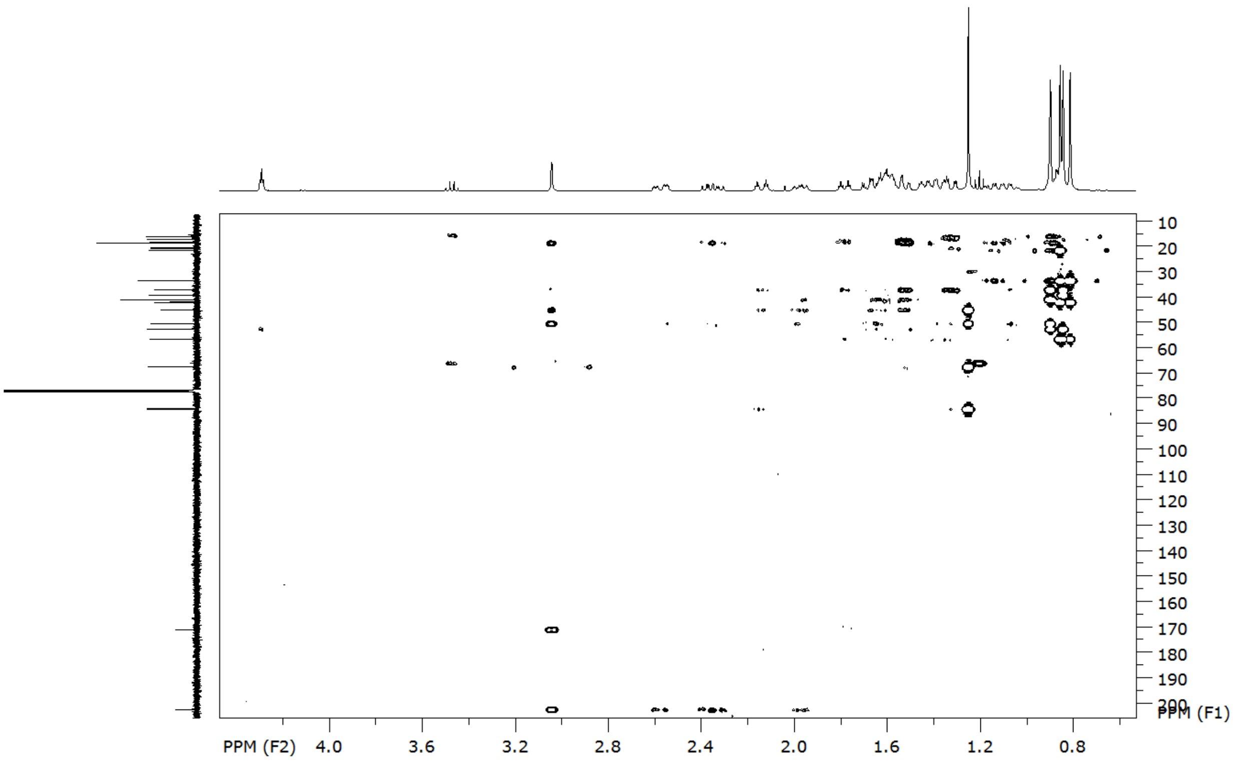

^1H, ^{13}C HMBC spectrum for compound **8**.

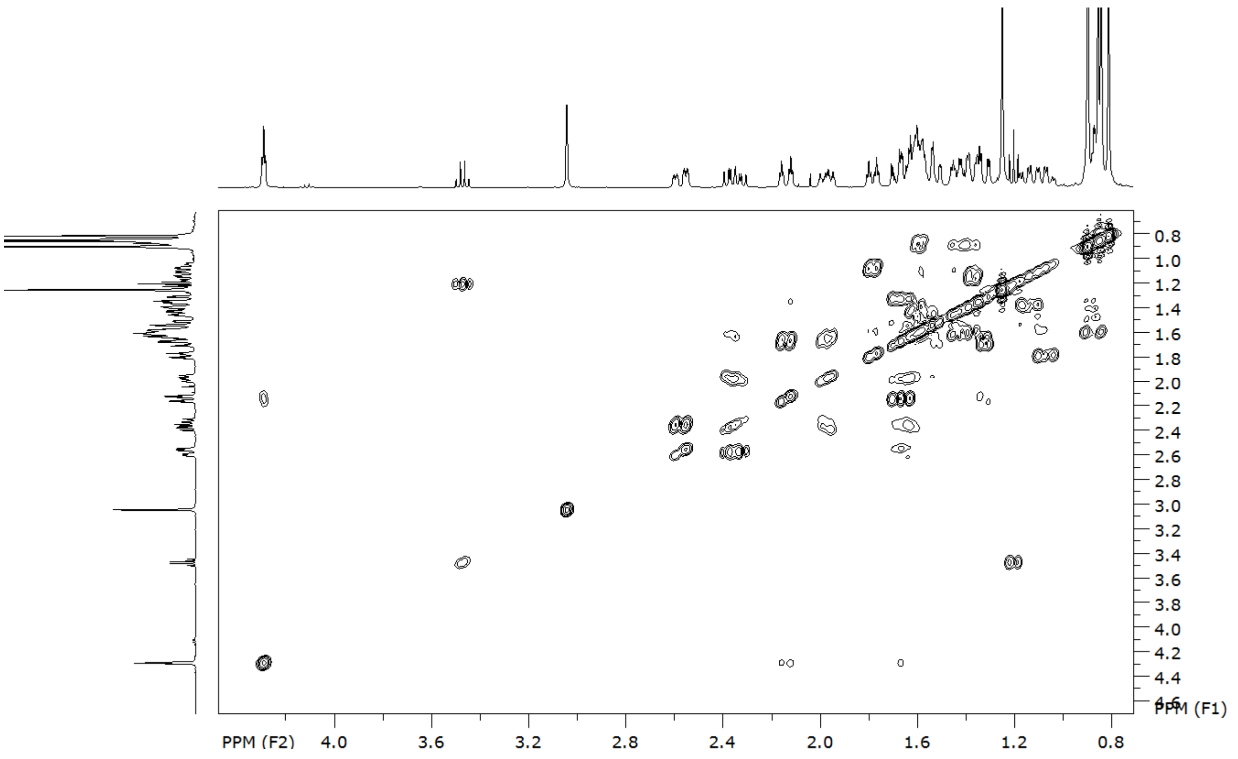

^1H-^1H COSY spectrum for compound **8**.

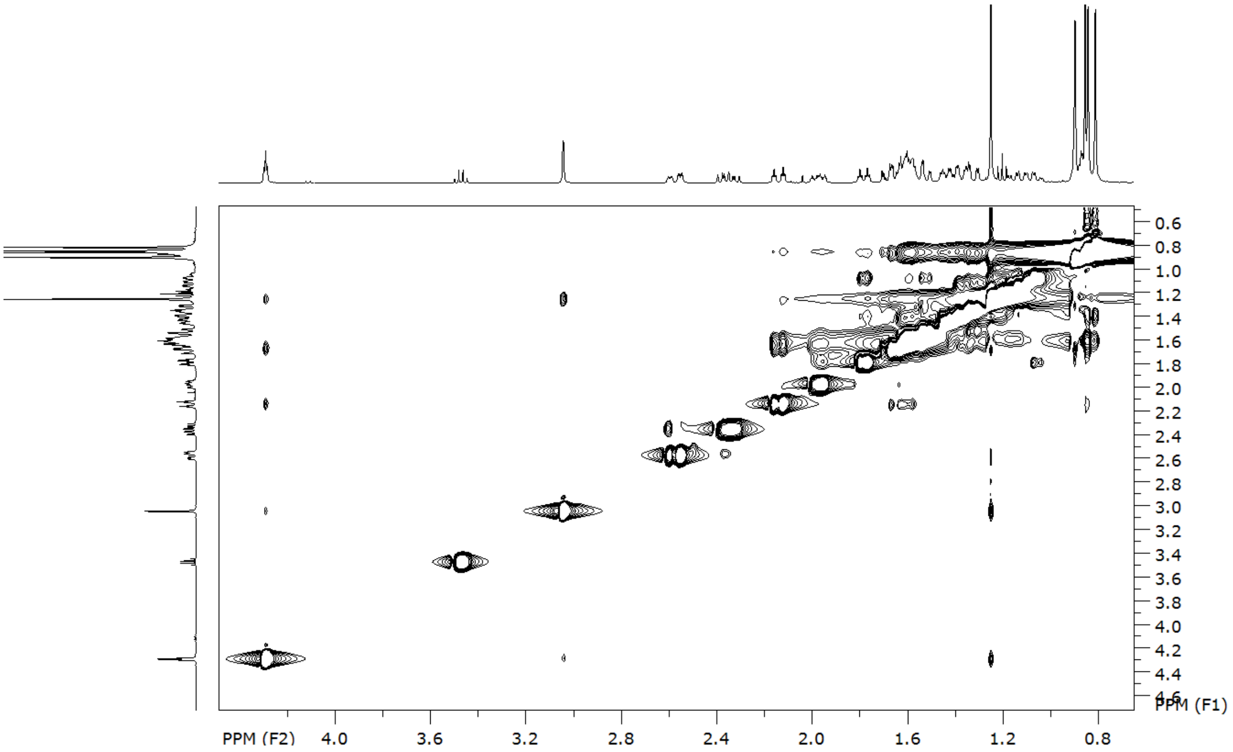

^{1}H-^{1}H NOESY spectrum for compound **8**.

REFERENCES

1. CrysAlisPro Software system, version 1.171.38.46, Rigaku Corporation: Oxford, UK, 2015.
2. Dolomanov, O.V.; Bourhis, L.J.; Gildea, R.J.; Howard, J.A.K.; Puschmann, H. OLEX2: a complete structure solution, refinement and analysis program. *J. Appl. Crystallogr*. **2009**, *42*, pp. 339–341. DOI:10.1107/S0021889808042726.
3. Sheldrick, G.M. SHELXT – Integrated space-group and crystalstructure determination. *Acta Crystallogr. Sect. A Foundations and Advaces* **2015**, *71* (1), pp. 3–8. DOI: 10.1107/S2053273314026370.
4. Sheldrick, G.M. Crystal structure refinement with SHELXL. *Acta Crystallogr. Sect.* C *Structural Chemistry* **2015**, *71* (1), pp. 3–8. DOI:10.1107/S2053229614024218.
